# Recent advances in the application of magnetic nanocatalysts in multicomponent reactions

**DOI:** 10.1039/d3ra01208e

**Published:** 2023-07-10

**Authors:** Hojat Veisi, Mozhgan Pirhayati, Pourya Mohammadi, Taiebeh Tamoradi, Saba Hemmati, Bikash Karmakar

**Affiliations:** a Department of Chemistry, Payame Noor University Tehran Iran hojatveisi@yahoo.com hojatveisi@pnu.ac.ir; b Department of Applied Chemistry, Faculty of Science, Malayer University Malayer Iran; c Production Technology Research Institute-ACECR Ahvaz Iran; d Department of Chemistry, Gobardanga Hindu College 24-Parganas (North) India bkarmakar@ghcollege.ac.in

## Abstract

Recently, the preparation and applications of magnetic nanostructures have attracted increasing attention in nanocatalysis studies, and magnetic nanoparticle (MNP) functionalized catalysts have been applied in important reactions such as Suzuki–Miyaura and Heck couplings. The modified nanocomposites demonstrate significant catalytic efficiency and excellent benefits in the context of catalyst recovery methods. This review discusses the recent modified magnetic nanocomposites in the field of catalytic applications along with the synthetic processes that are usually employed.

## Abstract 2

A comprehensive review on the preparation of diverse functionalized magnetic nanomaterials and their specific applications on multicomponent reactions.

## Graphical abstract

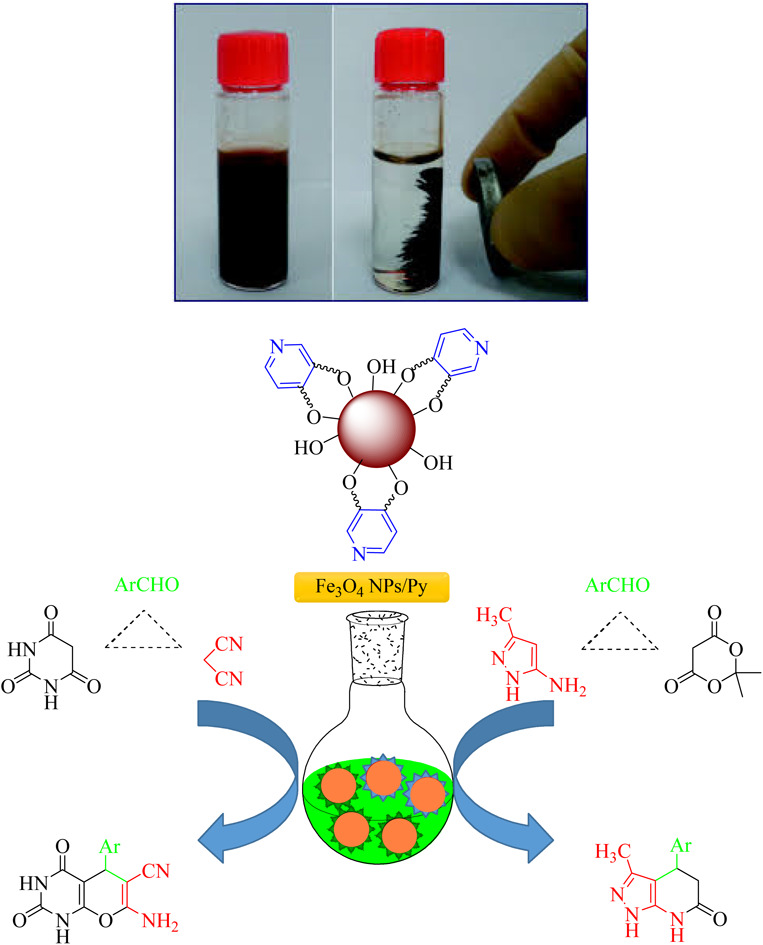

## 1. Introduction

Multicomponent reactions (MCRs) attract considerable attention in medicinal and organic chemistry owing to several advantages including their potential toward the production of desirable products with great atom economy. The reaction of three or more reactants in one step without the isolation of any intermediate and MCRs is an extremely useful method for the synthesis of complex organic compounds by easily available reactants.^1–9^ Synthetic researchers have repeatedly applied MCRs as a simple tool to construct a variety of molecules from bi-functional precursors that react sequentially in an intramolecular method.^10^

The challenge to create concise, conceptually fine, and new synthetic ways for multicomponent reactions has been a growing driving force in both industry and academia. Among the types of synthetic procedures, current studies have concentrated on establishing catalytic procedures from readily available starting materials under moderate reaction conditions.

A key part of “green chemistry” is the catalyst. Therefore, a stable and green catalyst must possess particular properties, such as great activity, low preparation cost, high selectivity, effective recovery, great stability, and excellent recyclability.^11^

Traditional catalysts can be classified into heterogeneous and homogeneous. The advantages of homogeneous catalysts include high selectivity and activity and available mechanistic investigations, leading to the optimization of catalysts. However, the problem of separation of these nanocatalysts from reaction media significantly limits their utilization in industry, particularly in the pharmaceutical and drug industry, due to the effect of metal pollution concerning metal-catalyzed production. The features, including the ease of handling, isolation, recycling, and being environmentally friendly, enable the application of heterogeneous catalysts to be more favorable. However, the activity of heterogeneous catalysts is generally less than that of homogeneous ones, owing to the smaller size of the interaction between the compounds and the surface of the catalyst.^12–18^

Therefore, it is necessary that a catalyst system, in addition to high selectivity and activity, can readily be isolated from the reaction media. This purpose can be achieved with nanocatalysts. Nano-sized catalysts have a high surface-to-volume ratio that is a suitable alternative to common catalysts.^19,20^ However, when the active site size is decreased to the nanoscale, the surface free energy rises considerably. This effect increases the aggregation of the particles into small clusters and demotes the catalytic performance. Moreover, separation and recovery of these catalysts become hard with decreasing size to nanoscale; in most cases, isolation by usual filtration can even become impossible.^21–24^

To resolve these problems, the application of magnetic nanoparticles (MNPs) seems to be the most reasonable solution.^25–30^ Their unique paramagnetic nature and intrinsic insolubility in most solvents let their easy and effective isolation from the reaction mixture by using a magnet. The catalysts can be consequently reutilized in another cycle. Recycling and reuse are two essential properties of magnetic nanocatalysts.^31–33^ Moreover, suitable surface-modification MNPs may be applied to prevent the aggregation of these nanoparticles, leading to stable, highly dispersed active particles. Due to these benefits, these functionalized nanoparticles have found broad applications in different fields, including magnetic resonance imaging,^34,35^ biomedicine,^36–39^ and hetero-catalysis.^40–47^ Hence, current improvements in magnetically recoverable catalysts (MRCs) have propelled their broad application in oxidation,^48–50^ hydrogenation,^51,52^ coupling reactions,^53–55^ cycloaddition,^56,57^ acylation,^58,59^ photocatalysis,^60–62^*etc.*

In recent years, several types of magnetic nanocatalysts have been used for the progress of multi-component reactions. In this review, we provide an overview of various kinds of magnetic nanocatalysts in MCRs and the main advantages of the reported methods.

## 2. Nano metal oxides (NMOs)

Between various nanocatalysts, nanocrystalline metal oxides are widely used in multi-component reactions. Among the metal oxide nanocatalysts, iron oxides (FeO, Fe_2_O_3_, and Fe_3_O_4_) are the most encouraging catalysts due to their ease of handling, recovery with an external magnet, high catalytic activities, in addition to the Lewis acid nature of iron that also catalyzes some of the organic reactions.^63–75^

**Scheme 1:**
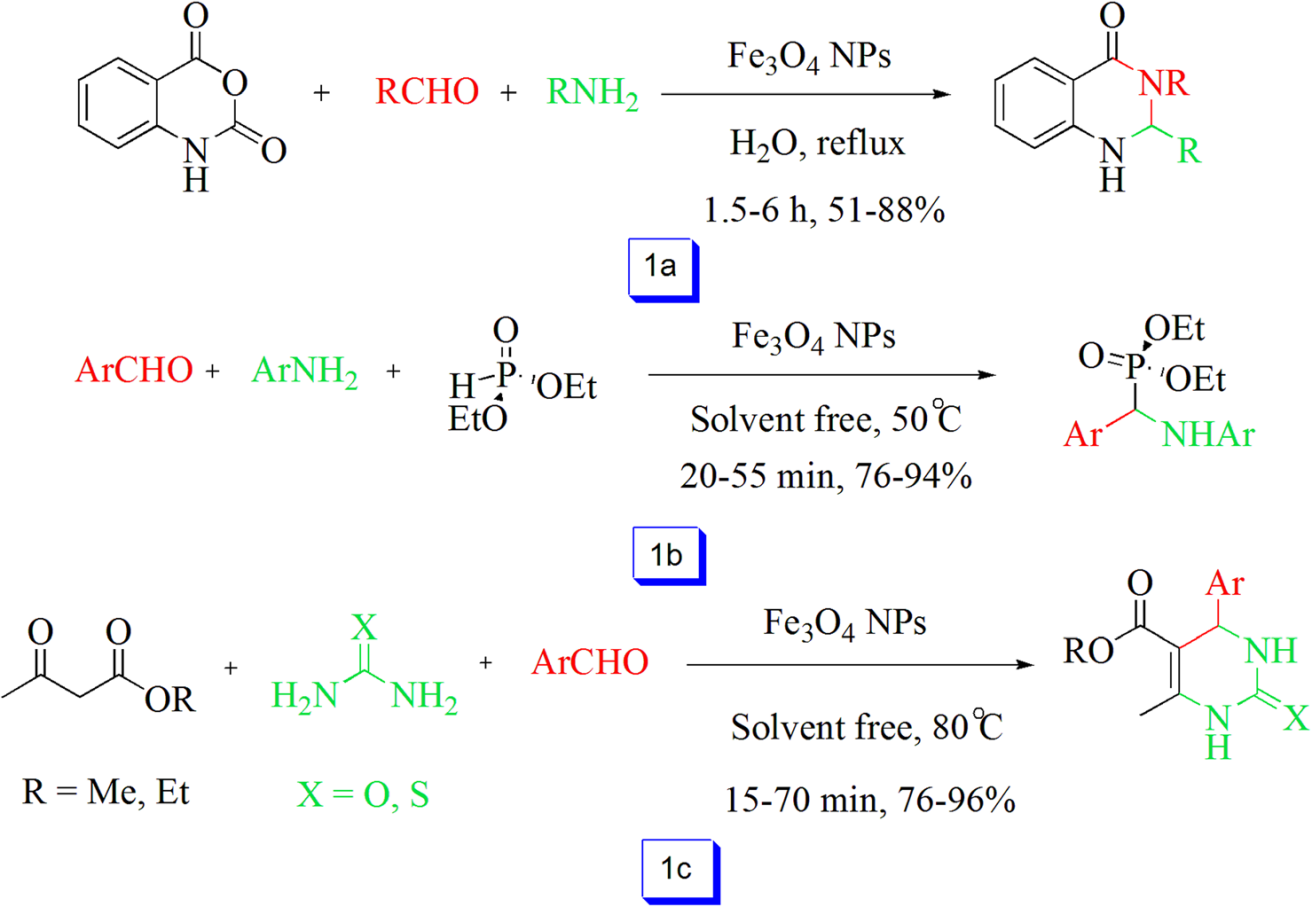
Synthesis of 2,3-dihydroquinazolin-4(1*H*)-ones in the presence of Fe_3_O_4_ NPs and H_2_O solvent at reflux condition (a), synthesis of α-aminophosphonates catalyzed by Fe_3_O_4_ NPs at 50 °C in solvent free conditions (b), and Fe_3_O_4_ NPs catalyzed the synthesis of dihydropyrimidinone derivatives at 80 °C in solvent free conditions (c).

Zhang *et al.*^76^ published an effective three-component condensation of aldehydes, isatoic anhydride, and amines in the presence of Fe_3_O_4_ under room temperature for a one-pot preparation of 2,3-dihydroquinazolin-4(1*H*)-ones. Utilizing this method, aromatic amines were observed to be efficient compounds and provided the products in high yields, and the reaction progressed smoothly when aliphatic amines were used (Scheme 1a). Additionally, the catalyst without a decrease in catalytic performance could be reutilized for up to five continuous cycles.

Reddy *et al.*^77^ used Fe_3_O_4_ nanoparticles for the preparation of α-aminophosphonates from the condensation of amines, aldehydes, and diethyl phosphate under solvent-free conditions. This reaction is appropriate for a large number of aldehydes, including aliphatic or aromatic, which react well and has excellent yields. Further, the catalyst was reutilized for ten runs without a considerable decrease in catalytic performance (Scheme 1b).

An effective procedure for the one-pot preparation of dihydropyrimidinones (thiones) in the present nanocatalyst was studied by Nasr-Esfahani *et al.*^78^ Results demonstrated that various types of aromatic aldehydes reacted well with other reagents to obtain the dihydropyrimidinone derivatives in solvent-free conditions with high yields. It is worth mentioning that aromatic aldehydes reacted in a lesser time compared with aliphatic aldehydes (Scheme 1c). The nanocatalyst could be recovered and reused effectively from the reaction mixture for at least four cycles without a noticeable loss in its catalytic efficiency.

**Scheme 2:**
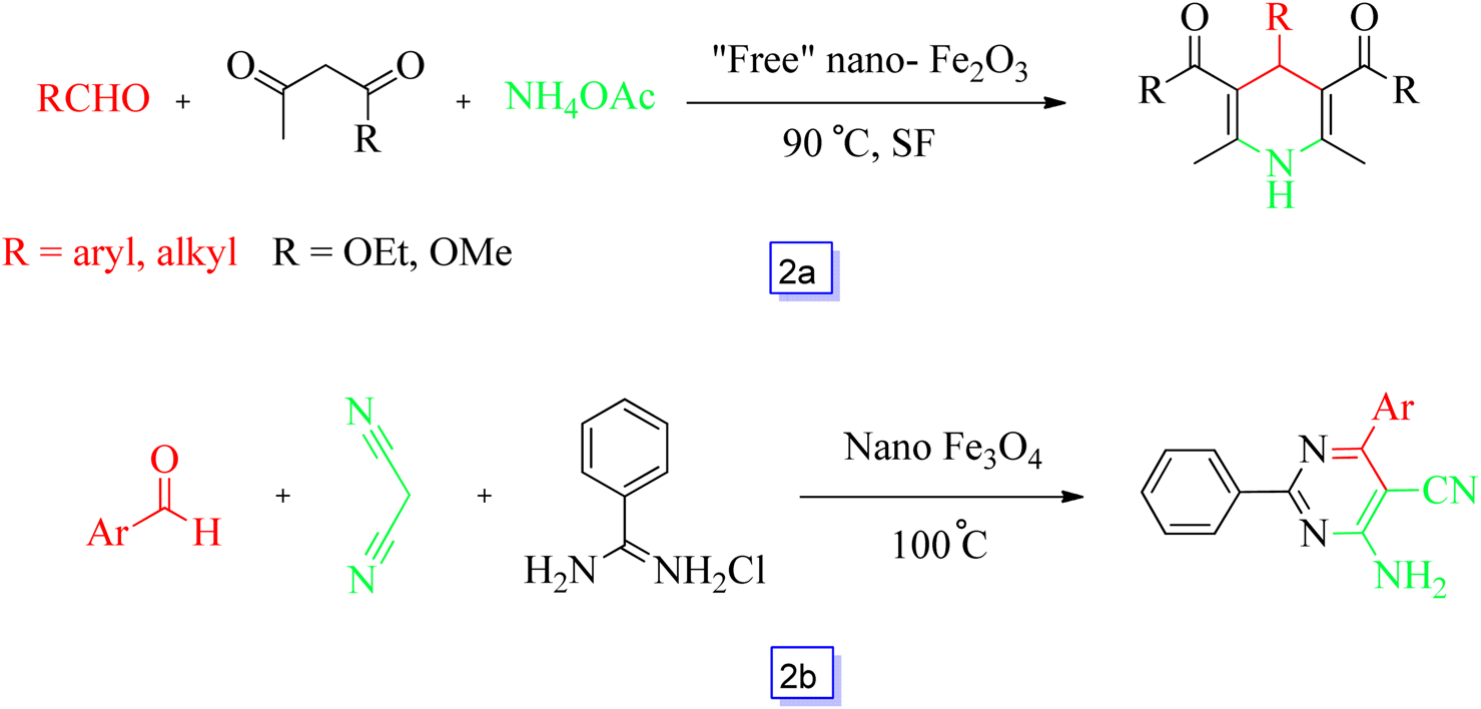
Synthesis of 1,4-dihydropyridines using Fe_2_O_3_ NPs at 90 °C (a) and reaction used for the synthesis of pyrimidine-5-carbonitrile derivatives at 100 °C (b).

Zolfigol, Khazae, Kolvari, *et al.*^79^ recently studied the preparation of Hantzsch 1,4-dihydropyridines compounds in the presence of free nano-Fe_2_O_3_ as a Lewis-acid catalyst. A large number of aliphatic, aromatic, and heteroaromatic aldehydes were exposed to react with β-keto compounds and ammonium acetate with good yields under moderate conditions. This eco-friendly nanocatalyst could be directly reutilized without any deactivation even after separation from the reaction mixture (Scheme 2a).

The Rostamizadeh group^80^ reported the one-pot synthesis of 4-amino-6-aryl-2-phenyl pyrimidine-5-carbonitrile derivatives *via* the three-component reaction of an aldehyde, benzamidine hydrochloride, and malononitrile in the presence of Fe_3_O_4_ magnetic nanoparticles (Scheme 2b). The products were synthesized with high yields in 1–1.5 h in solvent-free conditions. Under these conditions, aromatic aldehydes with electron-donor and electron-withdrawing substituents exhibited remarkable reactivity in this method. Additionally, these chemicals were appraised for biological performance and demonstrated antibacterial performance related to the reference penicillin.

## 3. Ferrites of MNPs catalysis

Other forms of iron oxide, spinel ferrites (MFe_2_O_4_), have also attracted much attention to MNPs catalysis due to unique properties such as environmental stability and ferrimagnetism. In MFe_2_O_4_ compounds, M is a transition element, such as Cu, Zn, Mn, Ni, and Co.

**Scheme 3:**
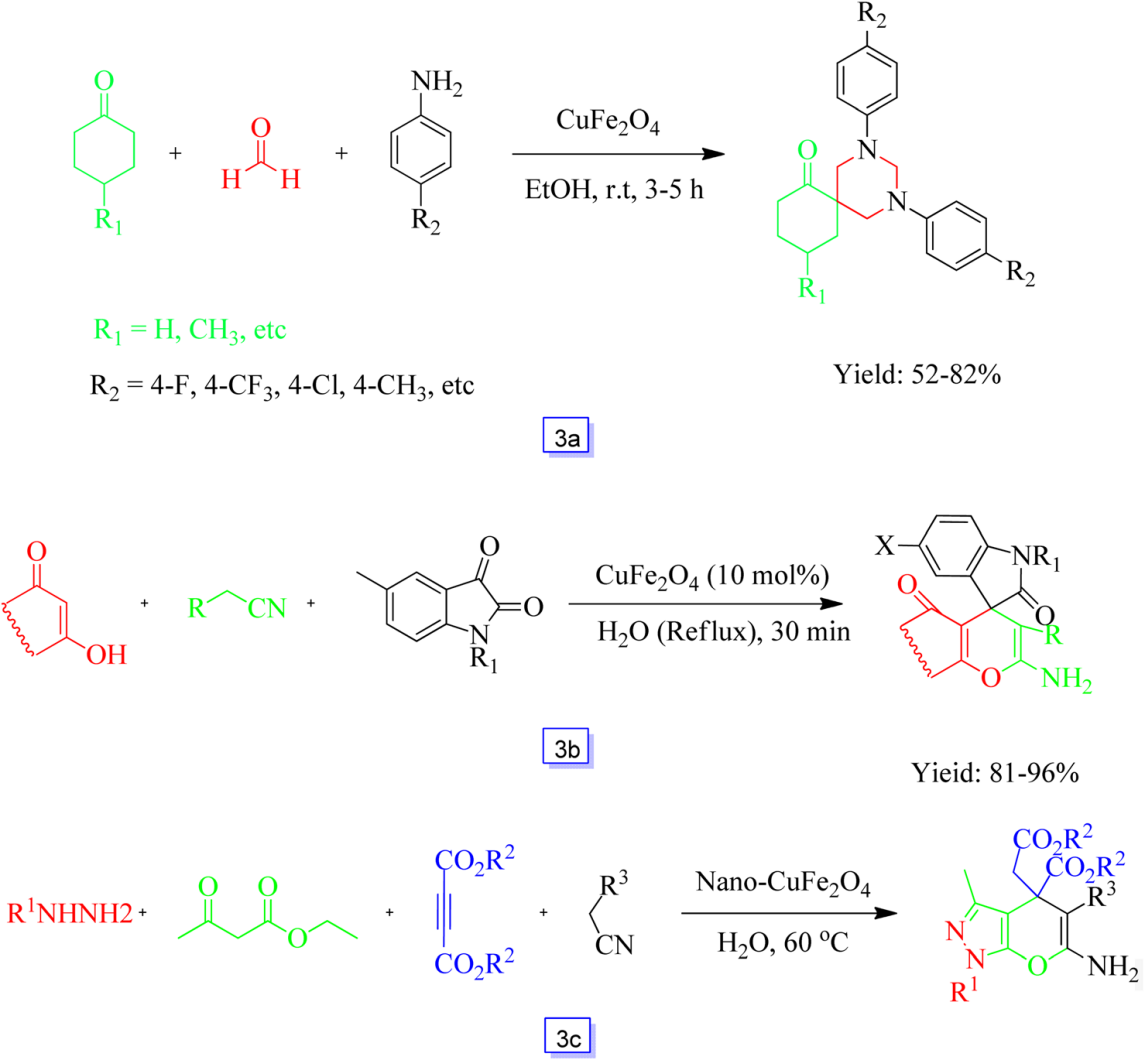
Synthesis of spirohexahydropyrimidines from ketones, aromatic amines, and formaldehyde catalyzed by CuFe_2_O_4_ at room temperature in ethanol solvent (a), synthesis of spiro[indolinepyrazolopyridopyrimidine] derivatives in the presence of CuFe_2_O_4_ and H_2_O solvent at reflux conditions (b) and four-component synthesis of 3-methyl-1,4-dihydropyrano[2,3-*c*]pyrazole derivatives in the presence of nano-CuFe_2_O_4_ and H_2_O solvent at 60 °C (c).

Dandia *et al.*^81^ showed that CuFe_2_O_4_ is a powerful and magnetic nanocatalyst for the one-pot preparation of spirohexahydropyrimidines through aromatic amines, formaldehyde, and ketones (Scheme 3a). The reaction was extended to several aromatic amines, and CuFe_2_O_4_ catalytic performance remained unchanged during five cycles, showing the effectiveness and “green” aspect of this nanocatalyst.

The catalytic property of a magnetic CuFe_2_O_4_ nanocomposite in the synthesis of spirooxindole *via* new multicomponent reactions was investigated by Ghahremanzadeh's group.^82^ The one-pot preparation of spirooxindole-fused heterocycles was achieved by cyanomethanes, cyclic 1,3-dicarbonyl derivatives, and isatins as reactants in the presence of a catalyst in H_2_O as the reaction solvent, obtaining spirooxindoles in 81% to 97% yields (Scheme 3b). In the primary examination, the reaction of malononitrile, 3-hydroxy-1*H*-phenalen-1-one, and isatin was performed in the presence of CuFe_2_O_4_ (10 mol%) at refluxing temperature for 0.5 h, and the intended spirooxindole was separated in 90% yield. It is important to note that after the first run, copper ferrite nanoparticles as a catalyst could be recovered and reutilized for four continuous cycles without a remarkable decrease in yield.

Pradhan and co-workers^83^ studied the preparation of dihydropyrano[2,3-*c*]pyrazole derivatives in one-pot at moderate reaction conditions and in high yields using CuFe_2_O_4_ nanoparticles as an effective nanocatalyst. The four-component reaction of a broad diversity of hydrazine derivatives, dialkyl acetylene dicarboxylates, ethyl acetoacetate, and alkyl nitrile derivatives, such as ethyl cyanoacetate and malononitrile, provided the desirable dihydropyrano[2,3-*c*]pyrazoles in excellent efficiency (Scheme 3c).

**Scheme 4:**
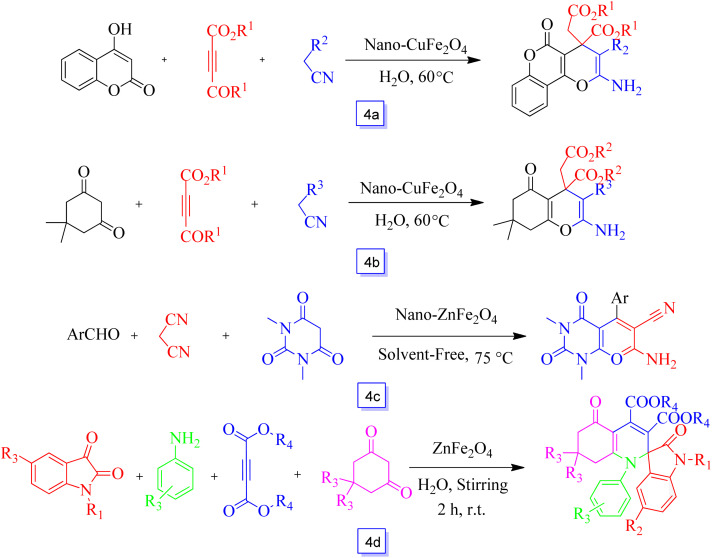
Synthesis of pyrano[3,2-*c*]coumarin and 4*H*-chromene derivatives in the presence of nano-CuFe_2_O_4_ and H_2_O solvent at 60 °C (a), synthesis of 4*H*-chromene derivatives in the presence of nano-CuFe_2_O_4_ and H_2_O solvent at 60 °C (b), synthesis of pyrano[2,3-*d*]pyrimidines in the presence of nano-ZnFe_2_O_4_ at 75 °C under solvent free conditions (c) and synthesis of spiro[indoline-3,2′-quinoline] derivatives in the presence of ZnFe_2_O_4_ and H_2_O solvent at room temperature (d).

Also, CuFe_2_O_4_ nanoparticles were used for the synthesis of pyrano[3,2-*c*]coumarin derivatives in one-pot at moderate reaction conditions in the aqueous media and in high yields.^83^ The reaction progresses through the MCR's of dialkyl acetylene dicarboxylates, 4-hydroxycoumarin, and ethyl cyanoacetate or malononitrile (Scheme 4a).

Catalytic recovery capability was investigated for the preparation of dihydropyrano[2,3-*c*]pyrazole. The results demonstrated that the nanocatalyst could be recovered in at least six cycles without a considerable decrease in efficiency.

CuFe_2_O_4_ magnetic nanoparticles were synthesized by Das *et al.*^84^ and used for the one-pot multi-component synthesis of 4*H*-chromene derivatives at moderate reaction conditions in aqueous media with high yields. The chemical reaction progresses through MCR's of cyclohexane-1,3-dione or dimedone, dialkyl acetylene dicarboxylates, and ethyl cyanoacetate or malononitrile (Scheme 4b).

Khazaei and colleagues^85^ reported the one-pot preparation of pyrano[2,3-*d*]pyrimidines *via* the three-component condensation of malononitrile, 1,3-diethyl barbituric acid, and aromatic aldehydes in the presence of heterogeneous ZnFe_2_O_4_ nanoparticles in solvent-free conditions (Scheme 4c). ZnFe_2_O_4_ nanoparticles as a Lewis acid (with the Fe^3+^ in Fe_2_O_3_) and the basic compound (related to the O^2−^ in ZnO) can catalyze this reaction. This procedure provides favorable products in good yields and in almost quick reaction times; it is noteworthy that aldehydes bearing electron-releasing groups increased the reaction times.

Pramanik and colleagues^86^ prepared a dual Lewis acid-base combined catalyst, *i.e.*, the ZnFe_2_O_4_ nanoparticles. For comparison of the catalytic activity of these catalysts, preparation of functionalized tetrahydrospiro[indoline-3,2′-quinoline] derivatives *via* the reaction of dialkyl acetylene dicarboxylates, arylamines, cyclohexane-1,3-diones, and isatin derivatives in aqueous media at ambient temperature was performed, providing products in high yields (Scheme 4d).

Ni–Zn ferrites are the most useable magnetic nanoparticles because they have high Curie temperature, high saturation magnetization, chemical stability, and high permeability.^87^

**Scheme 5:**
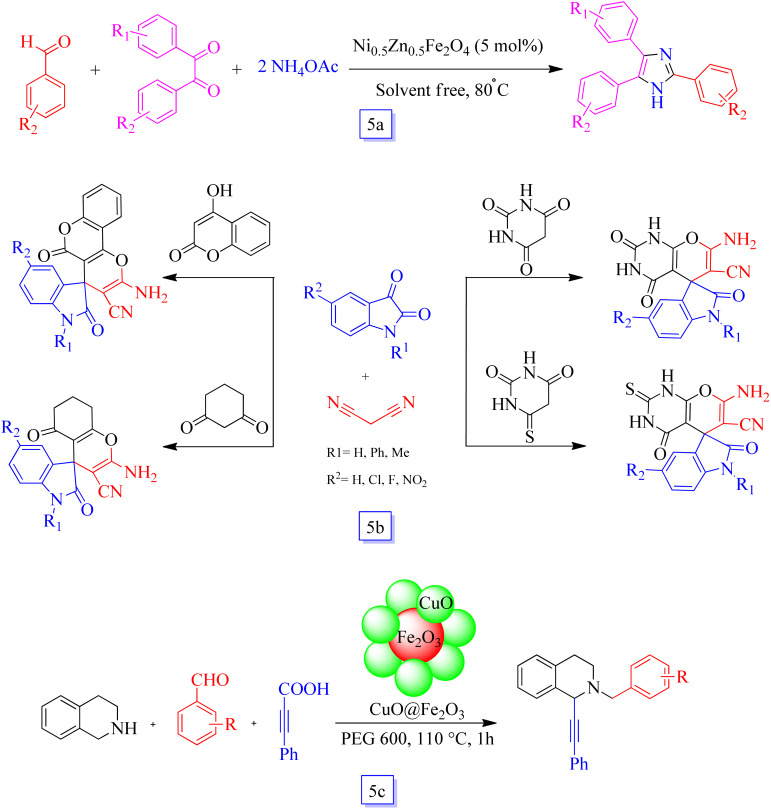
The synthesis of 2,4,5-triaryl imidazoles in the presence of Ni_0.5_Zn_0.5_Fe_2_O_4_ at 80 °C at reflux conditions (a), synthesis of spirooxindoles using magnetic nano-sized copper ferrite (b), and CuO@Fe_2_O_3_ MNP catalyzed C1-alkynylation of THIQ *via* decarboxylative A3 coupling reaction in the presence of Cu@Fe_2_O_3_ and PEG solvent at 110 °C (c).

Khazaei *et al.*^88^ studied a simple and effective process for the one-pot preparation of 2,4,5-triaryl substituted imidazoles using a three-component reaction in the presence of Ni_0.5_Zn_0.5_Fe_2_O_4_ as a reusable and heterogeneous nanocatalyst under solvent-free conditions (Scheme 5a). This method has several benefits, such as good yields, green reaction conditions, quick reaction times, easy handling, and proficiency of the catalyst. The magnetic Ni_0.5_Zn_0.5_Fe_2_O_4_ nanocatalyst was isolated from the reaction media *via* a magnet. The prepared nanocatalyst was recovered seven times in the subsequent reactions without any significant loss in the yield.

Khodabakhshi *et al.*^89^ reported a new application of a bimetal magnetic catalyst, *i.e.*, copper ferrite nanoparticles were used to prepare several new spirooxindoles by a one-pot three-component reaction including isatin with malononitrile and Michael donors (Scheme 5b).

In addition, this nanocatalyst was decanted from the reaction mixture using a magnet and effectively reutilized for subsequent cycles and retained its high performance in the fourth reaction run.

Rawat's group^90^ synthesized CuO@Fe_2_O_3_ nanocatalyst for C1-alkynylation of tetrahydroisoquinoline (THIQ) through A3 coupling among THIQ, alkynes, and aldehydes and its decarboxylative procedures *via* replacement of alkynes with phenyl propionic acid (Scheme 5c). The described catalytic method was found to be magnetically recoverable 5 times without a remarkable decrease in its performance, proposing a low *E*-factor and great atom economy.

## 4. Modification of MNPs

A common problem of MNPs is the fast aggregation to large clusters and the subsequent decrease of the unique features correlated with catalytic reactions due to their large surface energy, small interparticle distances, and the presence of van der Waals forces. To resolve this difficulty, MNPs modification utilizing appropriate stabilizer ligands or coating substances (such as small molecules, polymers, silica, ionic liquids, carbon, and metal or metal oxide nanoparticles) has been confirmed as the best solution now. Thus, the modified systems present active groups or reaction units for noncovalent or covalently grafting the active catalytic sites onto the covered MNPs to produce magnetically recyclable nanocatalysts.

### 4.1. Stabilizing ligands for modification of MNPs

**Scheme 6:**
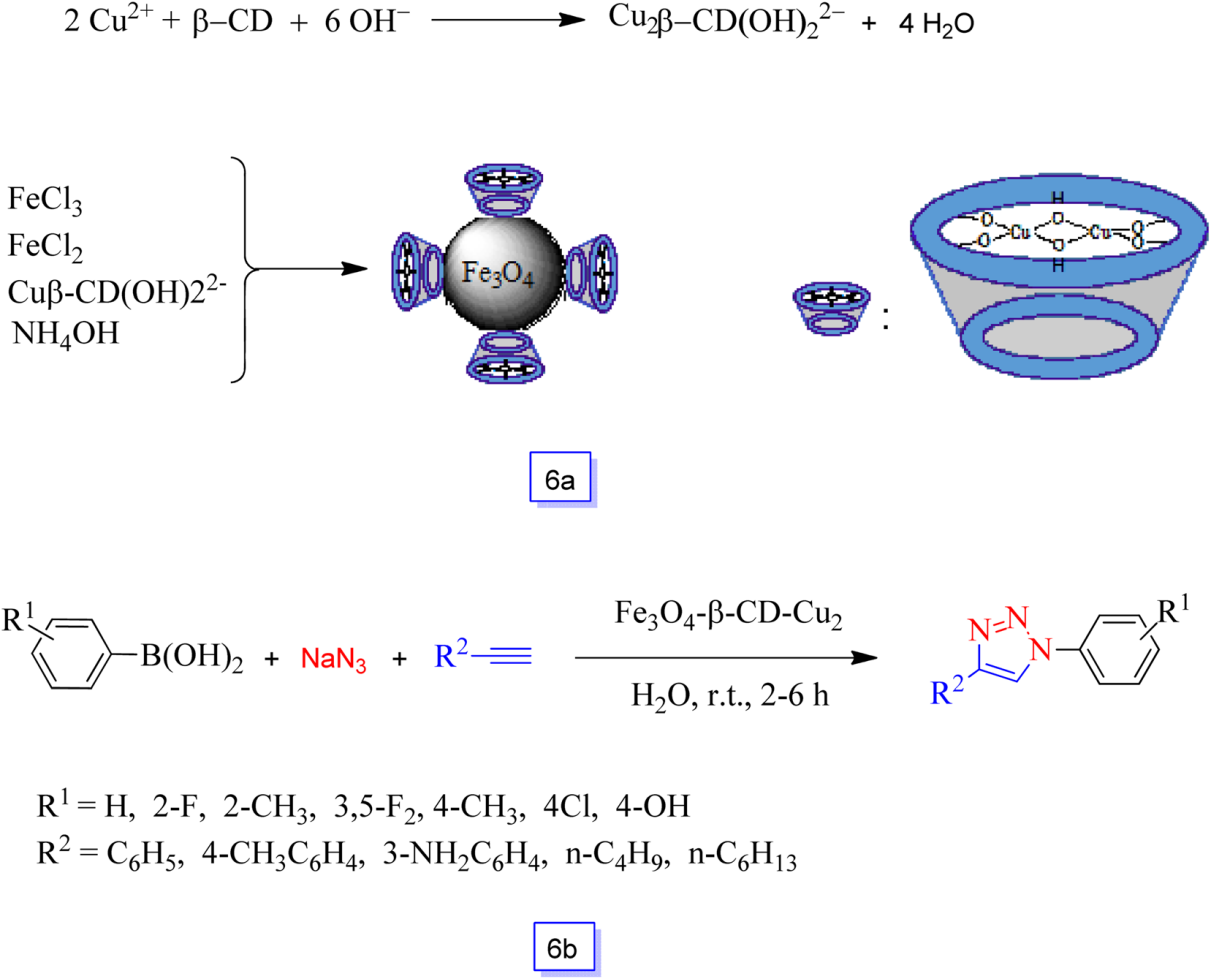
Preparation of the Fe_3_O_4_ magnetic nanoparticle-supported Cu(ii)–β-cyclodextrin complex catalyst (a) and one-pot synthesis using Fe_3_O_4_-supported Cu(ii)–β-cyclodextrin in the presence of Fe_3_O_4_–β-CD–Cu_2_ and H_2_O solvent at room temperature (b).

MNPs-supported binuclear Cu(ii)–β-cyclodextrin was readily synthesized *via* the addition of copper sulfate to the β-cyclodextrin in NaOH solution by Kaboudin and colleagues^91,92^ (Scheme 6a).

This catalyst was suitable for the preparation of 1,4-disubstituted 1,2,3-triazoles *via* one-pot *in situ* azidation of arylboronic acids and the subsequent click cyclization in aqueous solution in the air at room temperature (Scheme 6b). The results of the studies were successfully applied in the catalytic system producing high yields. In addition, Fe_3_O_4_–β-CD–Cu_2_ could be recycled by using a magnet and efficiently reutilized for 4 catalytic runs without a significant decrease in catalytic activity.

**Scheme 7:**
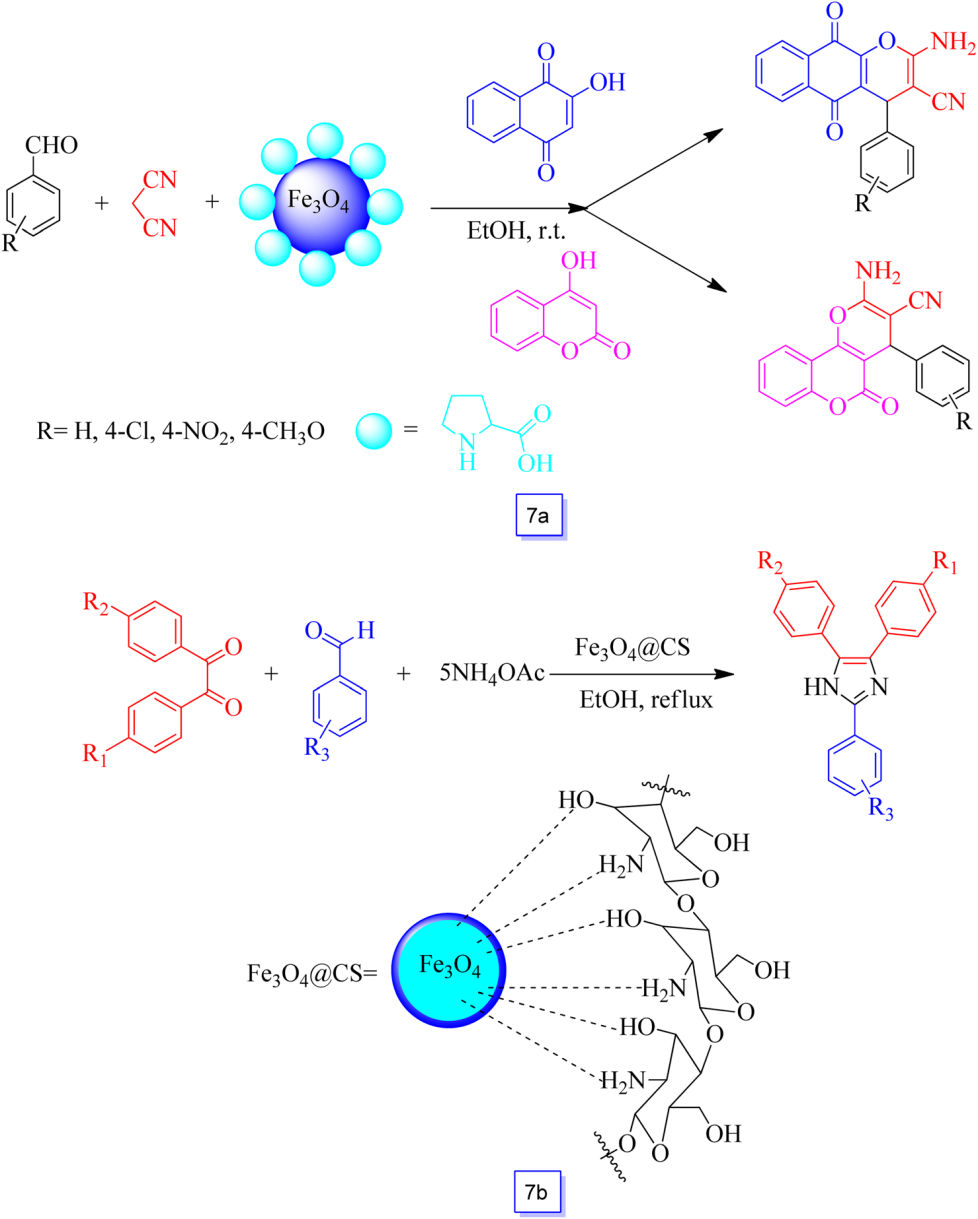
Synthesis of chromene derivatives using Fe_3_O_4_–proline MNPs in ethanol solvent at room temperature (a) and one-pot synthesis of 2,4,5-trisubstituted imidazoles catalyzed by Fe_3_O_4_@CS in the presence of ethanol solvent at reflux conditions (b).

Heydari *et al.*^93^ synthesized Fe_3_O_4_–proline MNPs without any supplemental linkers, and the catalytic efficiency of this nanocatalyst was considered in the preparation of chromene derivatives. Chromene derivatives are prepared through condensation of 2-hydroxynaphthalene-1,4-dione or 4-hydroxycoumarin, malononitrile, and aryl aldehyde in the presence of Fe_3_O_4_–proline nanoparticles at room temperature in good yields (Scheme 7a). Several derivatives of functionalized chromene were prepared under ambient conditions in high yields. The recoverability study showed that the application of a magnet provided separation of the nanocatalyst that was reutilized for a minimum of four runs without a decrease in performance and any iron leaching.

Safari and co-workers^94^ studied the synthesis of chitosan-coated Fe_3_O_4_ MNPs as renewable and heterogeneous magnetic biocatalysts and investigated their catalytic activity in the one-pot synthesis of 2,4,5-trisubstituted imidazoles through condensation of benzil derivatives, ammonium acetate, and aryl aldehydes in ethanol (Scheme 7b). The results showed good-to-high yields of the corresponding imidazoles. This heterogeneous bio-polymer catalyst was separated easily using a magnet, and the reusability of the synthesized nanocatalyst was favorably considered for six runs with only a very low decrease in catalytic performance.

**Scheme 8:**
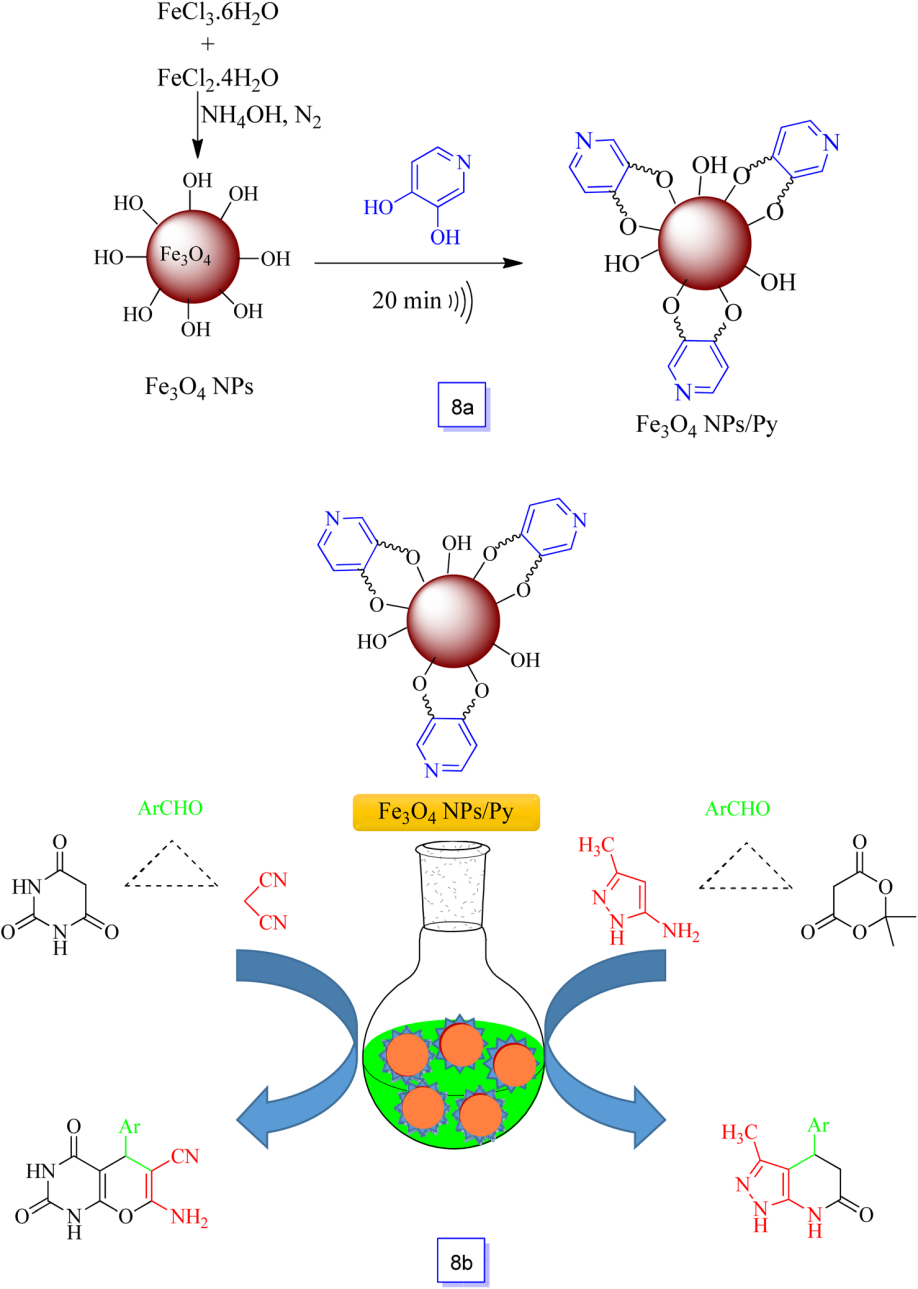
Preparation of Fe_3_O_4_/Py nanocatalyst (a) and a new nanocatalyst for one-pot synthesis of pyrazolo[3,4-*b*]pyridines and pyrano[2,3-*d*]pyrimidines (b).

In 2016, Veisi *et al.*^95^ additionally studied the synthesis of a new magnetic organocatalyst system, *i.e.*, 3,4-dihydroxypyridine supported on nano-Fe_3_O_4_ (Fe_3_O_4_/Py) (Scheme 8a).

This efficient nanocatalyst exhibited excellent catalytic performance in the domino condensation of different aromatic aldehydes, 5-methylpyrazol-3-amine and Meldrum's acid in moderate conditions and in ethanol as a solvent. Furthermore, the prepared nanocatalyst was found to be environmentally friendly with good catalytic activity for a three-component condensation of aromatic aldehydes with malononitrile and barbituric acid to the one-pot synthesis of 7-amino-2,4-dioxo-5-phenyl-2,3,4,5-tetrahydro-1*H*-pyrano[2,3-*d*]pyrimidine-6-carbonitriles (Scheme 8b). Moreover, noteworthy properties of this new catalyst method were the fast (within 15 s) and effective isolation of the nanocatalyst (100%) with a suitable external magnet, which minimized the loss of nanocatalyst during isolation and it could be reutilized for up to five runs (with insignificant Pd leaching) without notable loss in catalytic efficiency.

**Scheme 9:**
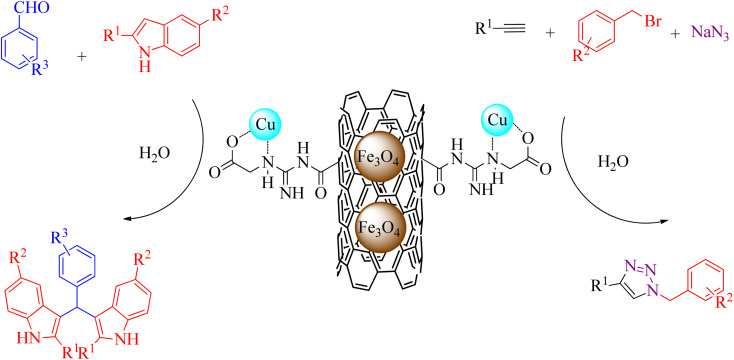
Synthesis of 1,2,3-triazole and bis(indolyl)methane derivatives using a Cu/MWCNT-GAA@Fe_3_O_4_ catalyst in the presence of H_2_O as a green solvent.

The synthesis of Cu-modified MWCNT-GAA@Fe_3_O_4_ nanocatalyst was reported by Shaabani *et al.*^96^ Cu/MWCNT-GAA@Fe_3_O_4_ demonstrated excellent catalytic performance for the preparation of 1,2,3-triazoles towards azide–alkyne 1,3-dipolar cycloaddition reactions and for the synthesis of bis(indolyl)methanes using condensation reaction in the presence of H_2_O as a green solvent (Scheme 9). The nanocatalyst was recyclable up to four cycles without much decrease in catalytic efficiency; the Cu leaching of the catalyst was very insignificant based on the FAAS analysis.

**Scheme 10:**
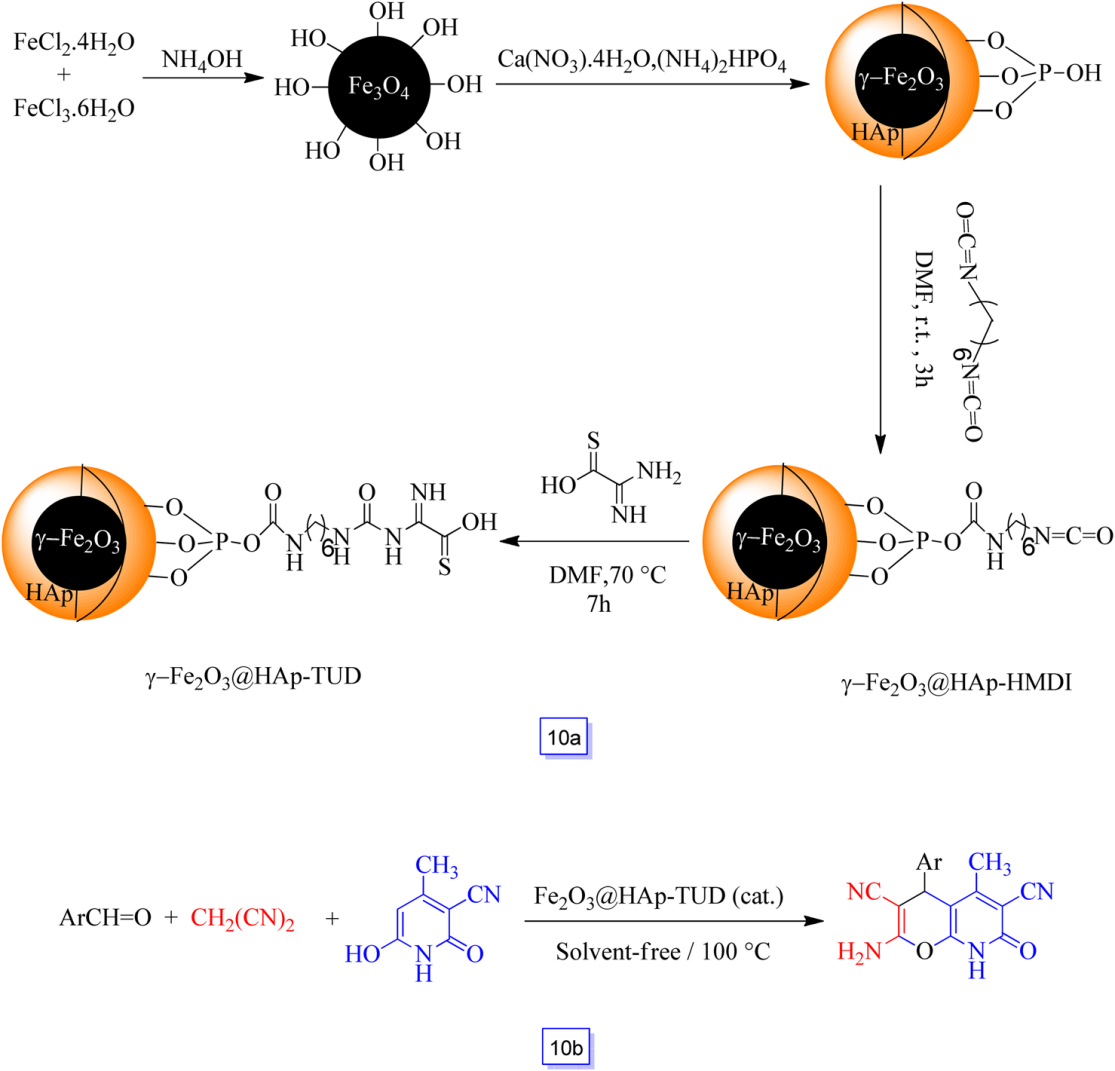
Preparation of the γ-Fe_2_O_3_@HAp-TUD MNPs (a) and γ-Fe_2_O_3_@HAp-TUD catalyzed synthesis of 2-amino-4-aryl-5-methyl-7-oxo-7,8-dihydro-4*H*-pyrano[2,3-*b*]pyridine-3,6-dicarbonitriles at 100 °C under solvent free conditions (b).

The synthesis of core–shell γ-Fe_2_O_3_@HAp-TUD, *i.e.*, thiourea dioxide-grafted hydroxyapatite-encapsulated hybrid nanoparticles, was reported by Azarifar and Ghaemi^97^ (Scheme 10a).

The catalytic activity of this newly prepared acidic nanocatalyst was considered *via* the one-pot three-component reaction between malononitrile, several substituted aldehydes, and 3-cyano-6-hydroxy-4-methyl-pyridin-2(1*H*)-one to provide the respective pyrano[2,3-*b*]pyridines in good yields (82–98%) in solvent-free conditions (Scheme 10b). It could be reused for seven cycles without any considerable decrease in catalytic efficiency. The aldehydes bearing electron-donating groups in comparison to electron-withdrawing ones, commonly react in longer times.

**Scheme 11:**
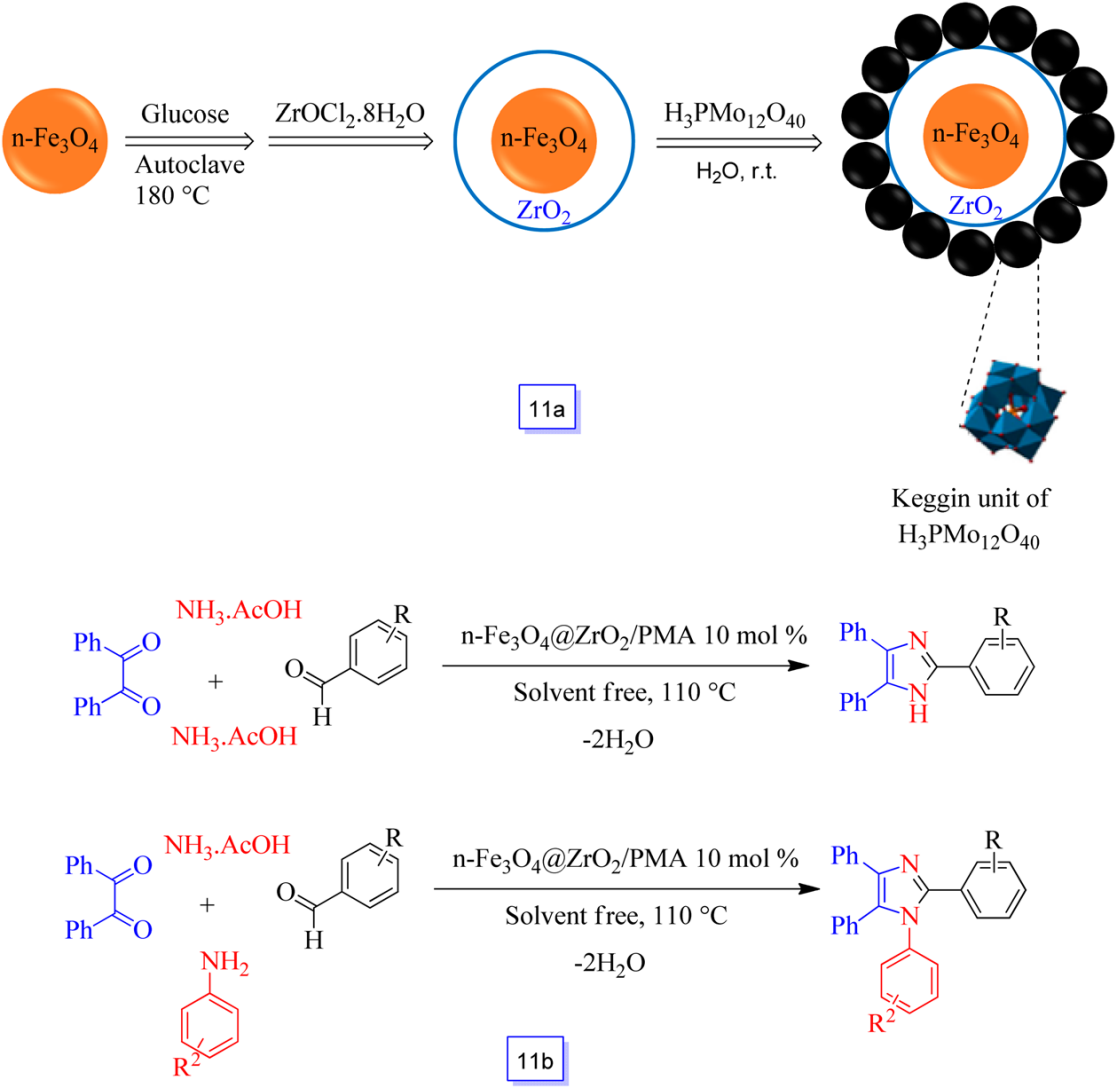
Preparation of *n*-Fe_3_O_4_@ZrO_2_ supported phosphomolybdic acid (a) and synthesis of 1,2,4,5-tetrasubstituted imidazoles and 2,4,5-trisubstituted imidazoles at 110 °C under solvent free conditions (b).

Kolvari *et al.*^98^ developed a heterogeneous nanosized acidic nanocatalyst called Fe_3_O_4_@ZrO_2_ supported PMA (or *n*-Fe_3_O_4_@ZrO_2_/PMA) so that the core–shell Fe_3_O_4_@ZrO_2_ surface was immobilized phosphomolybdic acid (H_3_PMo_12_O_40_) (Scheme 11a). This acidic nanocatalyst was used in several-component reactions for the one-pot preparation of multi-substituted imidazoles and for the preparation of 2,4,5-trisubstituted imidazoles from the reaction of benzil, aryl aldehydes, and NH_4_OAc, and also for the synthesis of 1,2,4,5-tetrasubstituted imidazoles from the condensation of aryl aldehydes, benzil, amines, and NH_4_OAc in solvent-free conditions (Scheme 11b).

It is noteworthy that the acidity content of the as-synthesized nanocatalyst demonstrated higher active sites related to homogeneous ones using potentiometric titration with *n*-butylamine. It is necessary to determine that this catalyst could be efficiently reutilized in the same reaction conditions for five continuous runs without a significant decrease in catalytic efficiency.

### 4.2. Nano porous systems

The favorite inorganic coating material for MNPs is silica due to its easy linking. Most of them were prepared in an organic solvent *via* hydrophobic capping agents that caused dispersibility in nonaqueous solvents but negligible dispersion in aqueous media. SiO_2_ as a covering shell increases biocompatibility and the water solubility of MNPs. The surface of SiO_2_ consists of two types of functional groups, [Si–O–Si and Si–OH], which provides functionalization with various functional groups. The functionalization of this surface using required reagents and linkers results in the production of a solid acidic catalyst.

**Scheme 12:**
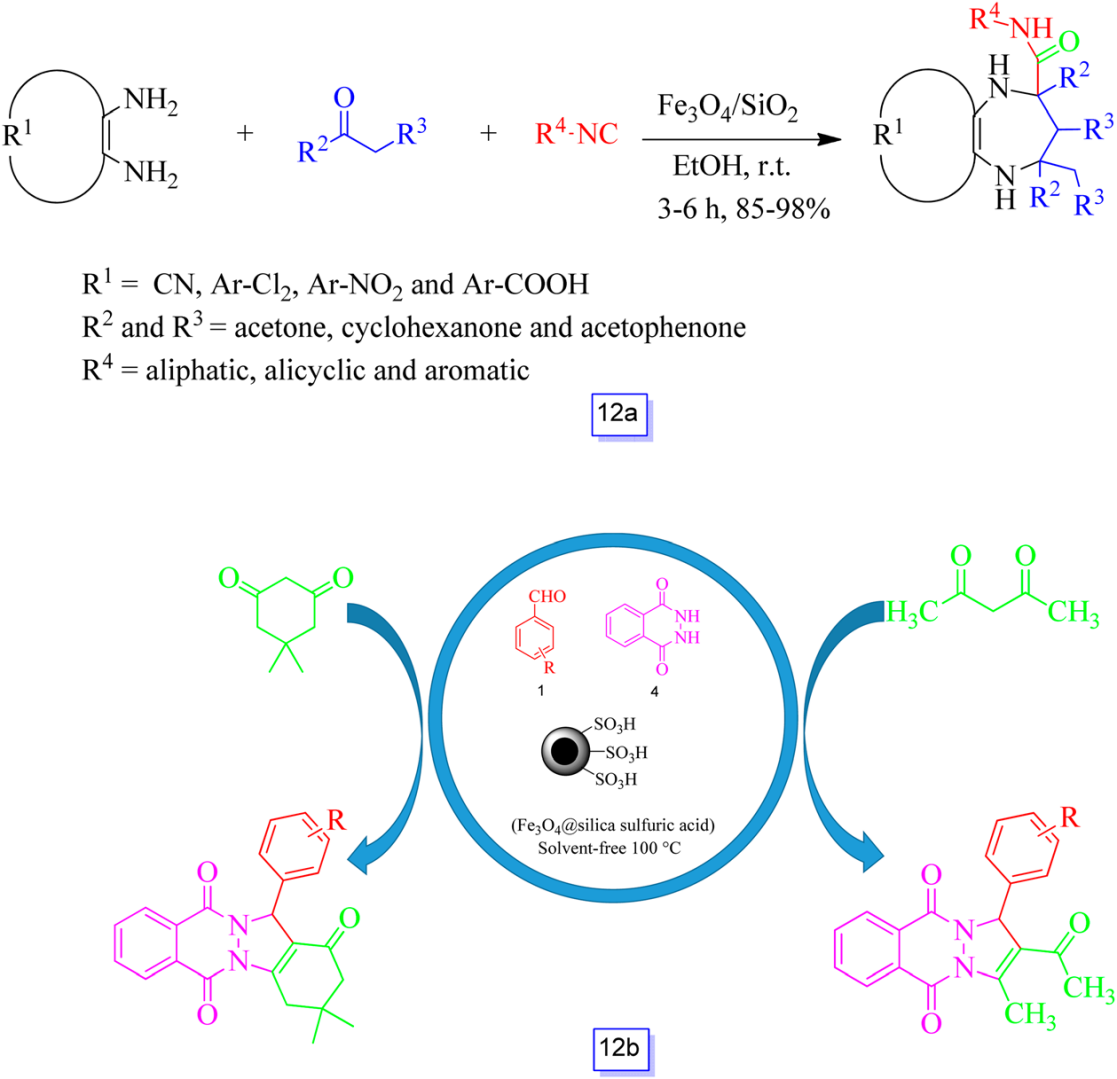
Synthesis of diazepine-2-carboxamide derivatives in the presence of Fe_3_O_4_/SiO_2_ nanocatalyst and ethanol at room temperature (a) and preparation of indazolo[2,1-*b*]phthalazine-triones and pyrazolo[1,2-*b*]phthalazine-diones catalyzed by Fe_3_O_4_@silica sulfuric acid at 100 °C under solvent free conditions (b).

Ali Maleki and co-workers^99^ also developed a novel protocol from ready and simple accessible precursors such as a 1,2-diamine, a cyclic or linear ketone, and an isocyanide for the one-pot multicomponent preparation of diazepine derivatives in the presence of Fe_3_O_4_/SiO_2_ as a magnetically recyclable nanocatalyst (Scheme 12a). In this method 4,5,6,7-tetrahydro-1*H*-1,4-diazepine-5-carboxamide derivatives and 2,3,4,5-tetrahydro-1*H*-1,5-benzodiazepine-2-carboxamide derivatives were synthesized with excellent yields and mild reaction conditions.

Kiasat and co-workers^100^ prepared a core–shell structure from sulfuric acid functionalized silica-coated magnetite nanostructure (Fe_3_O_4_@SiO_2_ sulfuric acid) (Scheme 12b) and investigated its catalytic activity for solid acid catalysts in the preparation of pyrazolo[1,2-*b*]phthalazine-diones and indazolo[2,1-*b*]phthalazine-triones by a one-pot and three-component reaction of aromatic aldehydes, phthalhydrazide, and linear or cyclic 1,3-diketones in solvent-free conditions. The advantages of this approach are a cleaner reaction, an easy procedure, application of a re-utilizable catalyst (for six runs), simple handling, and a multi-component reaction.

**Scheme 13:**
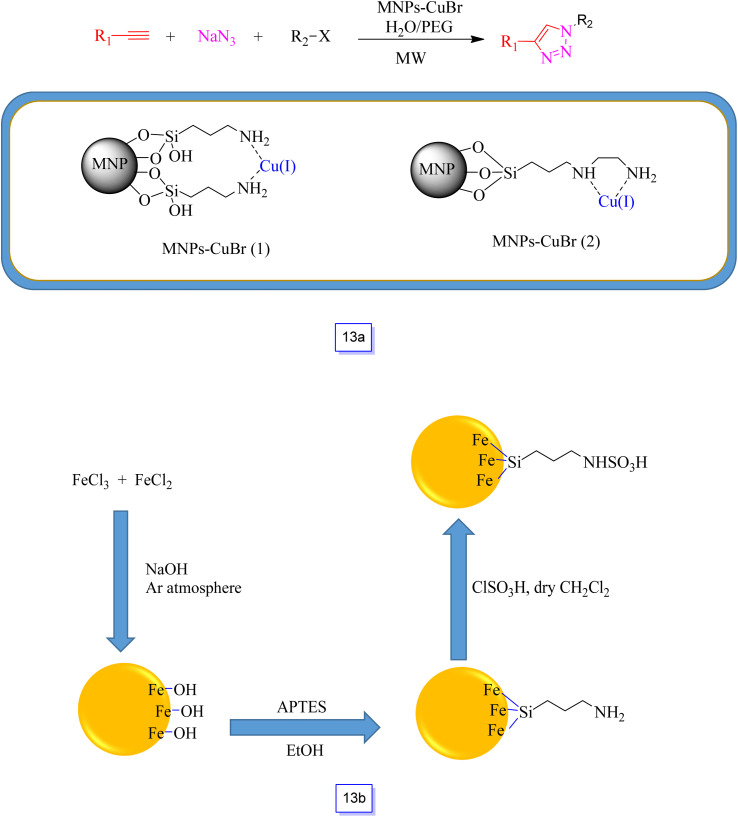
MNP-CuBr-catalyzed one-pot synthesis of 1,4-disubstituted 1,2,3-triazoles (a) and preparation steps for fabricating sulfamic acid-functionalized magnetic Fe_3_O_4_ nanoparticles (b).

Recyclable Fe_3_O_4_–immobilized copper(i) was readily prepared by Xiong *et al.*^101^ and they directly modified Fe_3_O_4_ MNPs with (3-aminopropyl)-trimethoxysilane and [3-(2-aminoethylamino)propyl]trimethoxysilane through a post-grafting process, followed by complexation with CuBr to produce MNPs-CuBr (1) and MNPs-CuBr (2), respectively (Scheme 13a). This catalyst was applied in one-pot multi-component azide–alkyne cycloaddition (CuAAC) synthesis from the reaction of sodium azide and benzyl chloride with phenylacetylene under microwave irradiation in the aqueous medium. As discussed in the presented research, microwave irradiation could significantly decrease the reaction time and concurrently improve the yields in comparison with common protocols. The first results of the investigation displayed that MNPs-CuBr (1) was a more effective catalyst than MNPs-CuBr (2), several halides, and terminal alkynes and confirmed its versatility in the presence MNPs-CuBr (1). Most of the 1,4-disubstituted 1,2,3-triazoles were separated in good to high efficiency with high selectivity. Moreover, the magnetically isolated nanocatalyst MNPs-CuBr (1) was simply recycled and reutilized for about seven runs with an inconsiderable loss of catalytic performance.

Safari and colleagues^102^ reported that on an amino-functionalized Fe_3_O_4_ nanoparticle surface, chlorosulfuric acid could be immobilized. The preparation of this material includes the operation of Fe_3_O_4_ with 3-aminopropyltriethoxysilane (APTES) through a silanization reaction, followed by the reaction with chlorosulfuric acid (Scheme 13b).

**Scheme 14:**
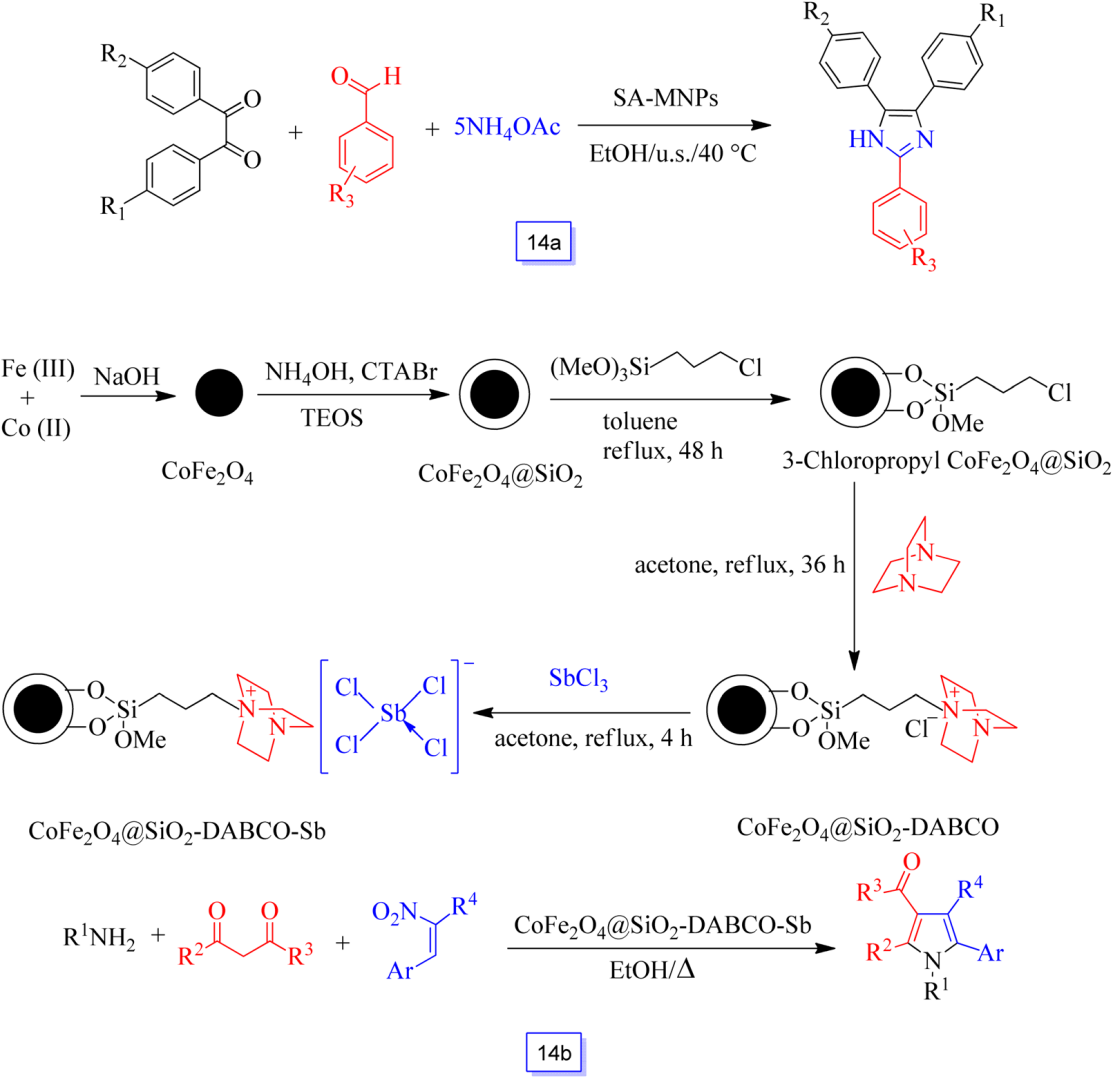
One-pot synthesis of 2-aryl-4,5-diphenyl imidazoles catalyzed by SAMNPs under ultrasound irradiation at ambient temperature in ethanol solvent (a) and synthesis of CoFe_2_O_4_@SiO_2_-DABCO-Sb and functionalized pyrroles (b).

This acid catalyst (SA-MNPs) was then tested for the one-pot synthesis of 2,4,5-trisubstituted imidazoles through a three-component condensation of aldehyde and NH_4_OAc with 1,2-diketone under sonication (Scheme 14a). This new heterogeneous catalyst also has the capability to sustain a broad type of substitution in the reagents. This nanocatalyst was recycled from the reaction mixture using a magnet, and the recovered catalyst could be applied in five continuous cycles without any remarkable decrease in efficiency.

**Scheme 15:**
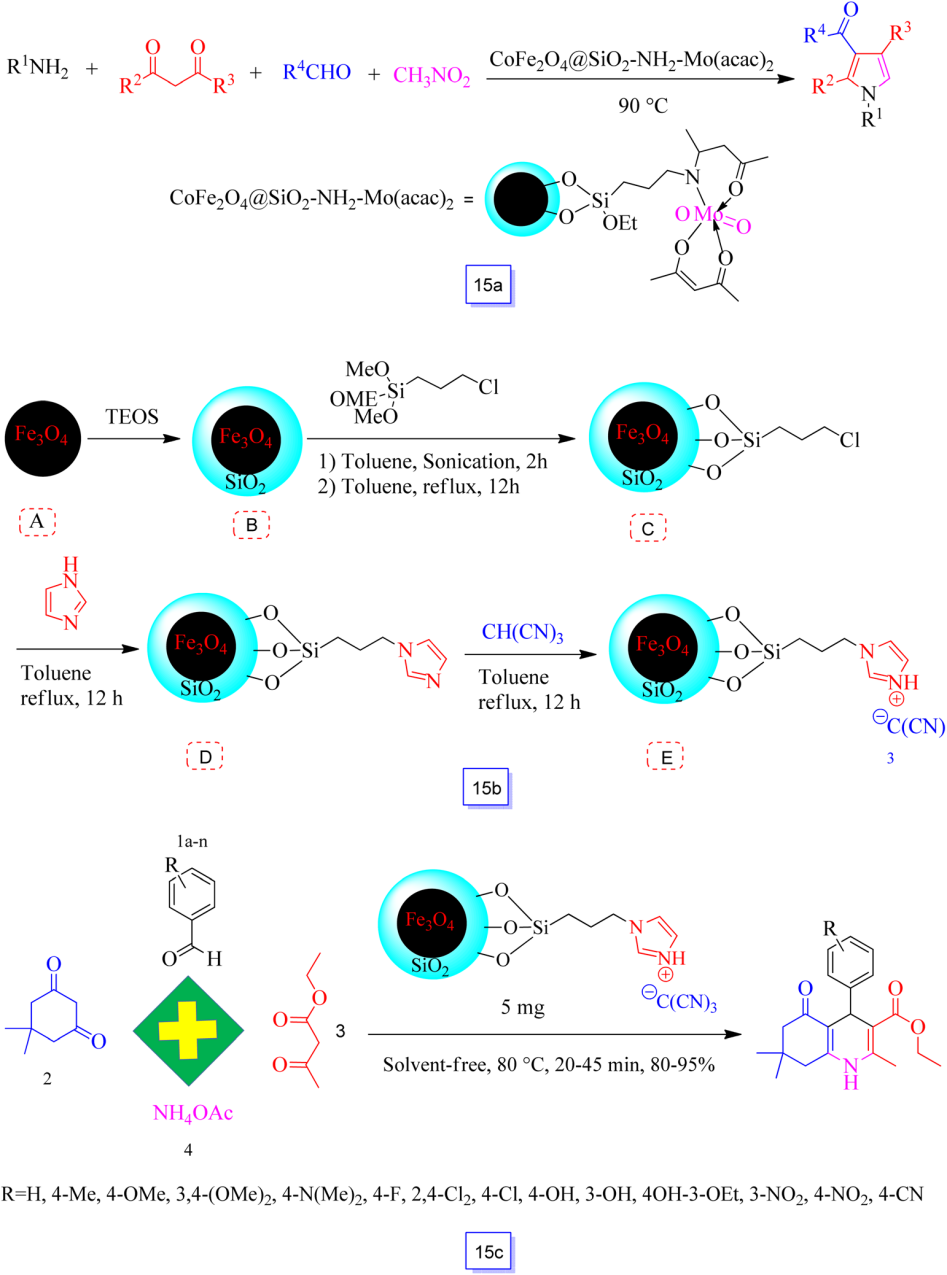
Synthesis of functionalized pyrroles in the presence of CoFe_2_O_4_@SiO_2_-NH_2_-Mo(acac)_2_ at 90 °C (a), preparation of{Fe_3_O_4_@SiO_2_@(CH_2_)_3_Im}C(CN)_3_ (b), and synthesis of polyhydroquinoline derivatives in the presence of MNPs@IL at 80 °C under solvent free conditions (c).

Multi-substituted pyrroles were synthesized in the presence of CoFe_2_O_4_ magnetic nanoparticle-supported Sb ([CoFe_2_O_4_@SiO_2_-DABCO-Sb]) as an effective nanocatalyst (Scheme 15) by Li *et al.*^103^ 4-*H*-Pyrans were prepared by the reaction of amines, nitroolefins, and 1,3-dicarbonyl compounds in good yields in short times. Furthermore, a variety of structurally diverse salicylaldehydes reacted favorably with active nucleophilic reagents to produce favorable coumarins in good to excellent yields. This CoFe_2_O_4_ nanoparticle-supported Sb catalyst was recycled after five continuous runs without a decrease in its catalytic performance.

Zhang and co-workers^104^ prepared a CoFe_2_O_4_ magnetic nanoparticle-supported molybdenum catalyst ([CoFe_2_O_4_@SiO_2_-PrNH_2_-Mo(acac)_2_]), which demonstrated a promising catalytic performance for the one-pot synthesis of multi-substituted pyrroles *via* reaction of the four-component reaction of amines, aldehydes, nitromethane, and 1,3-dicarbonyl compounds (Scheme 15a). This catalyst could be readily recycled by using a magnet and reutilized five cycles without a notable decrease in activity.^38^

Recent studies have shown that magnetic nanostructures can act as unique supports for ionic liquids (ILs) owing to their excellent stability, simple synthesis and high applicability, large surface area and simple isolation, and low toxicity and low cost.^105^

Zolfigol *et al.*^105^ demonstrated the stabilization of ionic liquid on silica coated on Fe_3_O_4_ surface {Fe_3_O_4_@SiO_2_@(CH_2_)_3_Im}C(CN)_3_ as a new heterogeneous catalyst for the preparation of polyhydroquinoline derivatives using the condensation of dimedone, ethyl acetoacetate, ammonium acetate, and a wide range of aryl aldehydes under green, mild, and solvent-free conditions (Scheme 15b and c).

All starting materials (aryl aldehydes such as those bearing electron-withdrawing and electron-donating groups and halogens) reacted with each other using catalyst MNPs@IL to produce the intended products in good yields in shorter reaction times. The nanocatalyst was reutilized for eight cycles. The obtained results show that the catalytic efficiency of this catalyst was recovered without any significant variations in the performance.

**Scheme 16:**
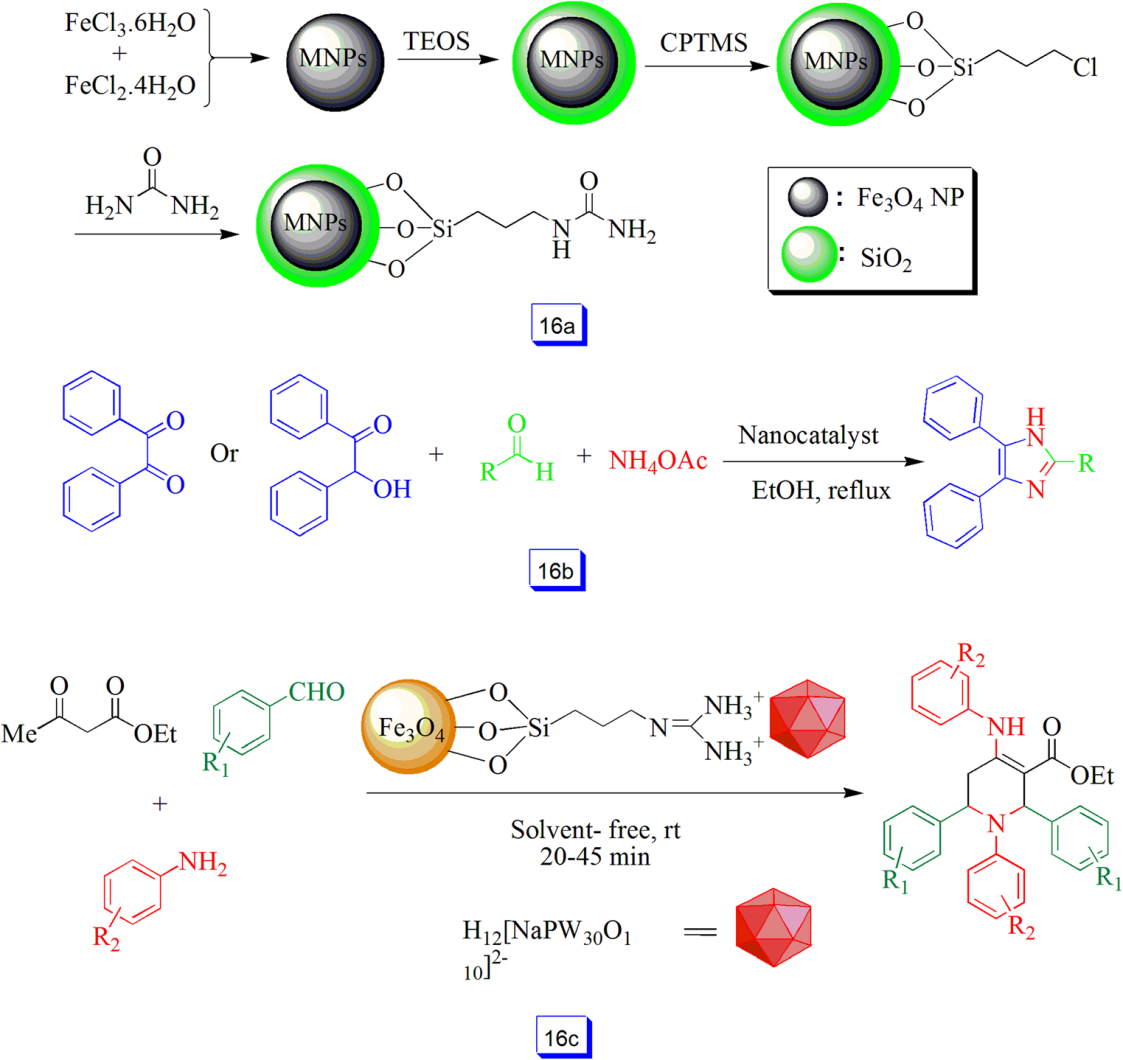
Preparation of Fe_3_O_4_/SiO_2_–urea nanoparticles (a), three-component reaction by Fe_3_O_4_/SiO_2_/urea and ethanol solvent under reflux conditions (b), and synthesis of tetrahydropyridine derivatives in the presence of Fe@Si-Gu-Prs at room temperature under solvent free conditions (c).

Maleki *et al.*^106^ reported that MNP-urea nanostructure could be applied as a new magnetic nanocatalyst for the preparation of substituted imidazoles (Scheme 16a). The compounds were prepared in high yield through the three-component reaction of benzoin or benzil and differently substituted aldehydes with ammonium acetate in the one-pot procedure to produce the intended imidazoles in the presence of Fe_3_O_4_/SiO_2_–urea nanocatalyst under refluxing conditions (Scheme 16b). To study the recyclability of this catalyst, after the end of the reaction, the nanocatalyst was easily isolated from the reaction mixture, washed, air-dried, and reused for a minimum of five runs. The reusability of the catalyst without significant loss in catalytic activity shows excellent and practical recoverability.

The chemical immobilization of Preyssler heteropolyacid (H_14_ [NaP_5_W_30_O_110_]) onto the Fe_3_O_4_ nanoparticles surface modified with guanidine-propyl-trimethoxysilane linker as an organic–inorganic hybrid nanocatalyst (Fe@Si-Gu-Prs) was achieved by Eshghi and co-workers^107^ and successfully used for the synthesis tetrahydropyridine. Therefore, the reaction of amines, aldehydes, and ethyl acetoacetate at ambient temperature and under solvent-free conditions provided excellent performance (Scheme 16c). A wide range of amines and aldehydes with substituents, either electron-withdrawing or electron-donating groups, favorably reacted *via* this procedure, and products were obtained in high yields (higher than 90%) following short reaction times. The nanocatalyst was recovered and reused for 5 cycles without any loss of catalytic efficiency.

**Scheme 17:**
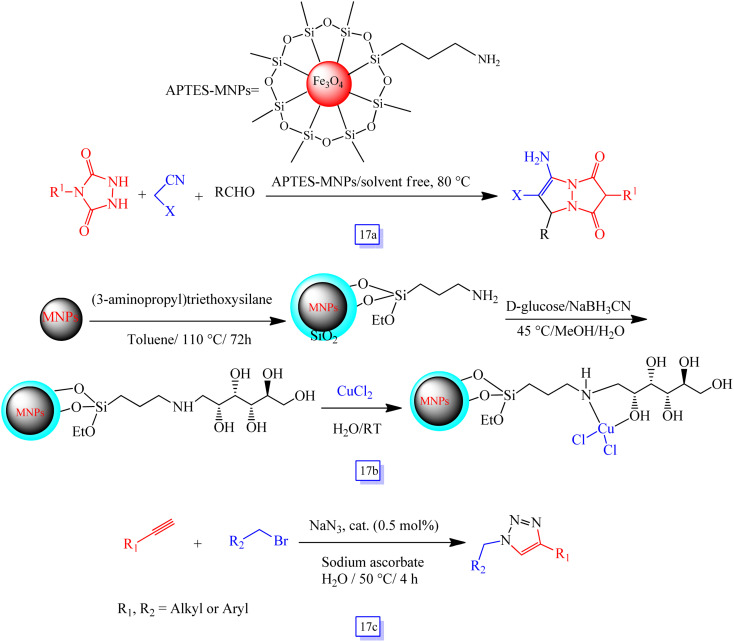
Synthesis of 7-amino-1,3-dioxo-1,2,3,5-tetrahydropyrazolo[1,2-*a*][1,2,4]triazole derivatives in the presence of APTES-MNPs at 80 °C under solvent free conditions (a), immobilization of glucose on Fe_3_O_4_@SiO_2_ (b), and Huisgen reaction catalyzed by [MNPs@GLU][Cl] in H_2_O solvent at 50 °C (c).

Shaterian and Moradi^108^ reported a simple procedure for the one-pot preparation of 7-amino-1,3-dioxo-1,2,3,5-tetrahydropyrazolo[1,2-*a*][1,2,4]triazole derivatives from the three-component reaction of 4-phenylurazole, aryl aldehydes, and malononitrile by using (3-aminopropyl)-triethoxysilane supported on Fe_3_O_4_ surface as a nanocatalyst (Scheme 17a). In addition, the nanocatalyst could be recovered several times without any appreciable loss in the yield.

Saberi *et al.*^109^ represented the stabilization of glucose onto nanoparticles of Fe_3_O_4_–silica coated@functionalized(3-aminopropyl)triethoxysilane to immobilize copper salts (Scheme 17b). They studied its utilization as a new magnetic nanocatalyst in the one-pot synthesis of 1,3-dipolar cycloaddition of phenylacetylene to azides in H_2_O, as a green solvent, through a three-component reaction of alkyl halides, alkynes, and sodium azide (Scheme 17c). The main advantage of this approach is the use of glucose as an efficient and green ligand that causes the catalyst to disperse in water by forming hydrogen bonds. Next, the catalyst was almost completely separated using an external magnet, and it was applied for seven further runs without loss in catalytic activity.

**Scheme 18:**
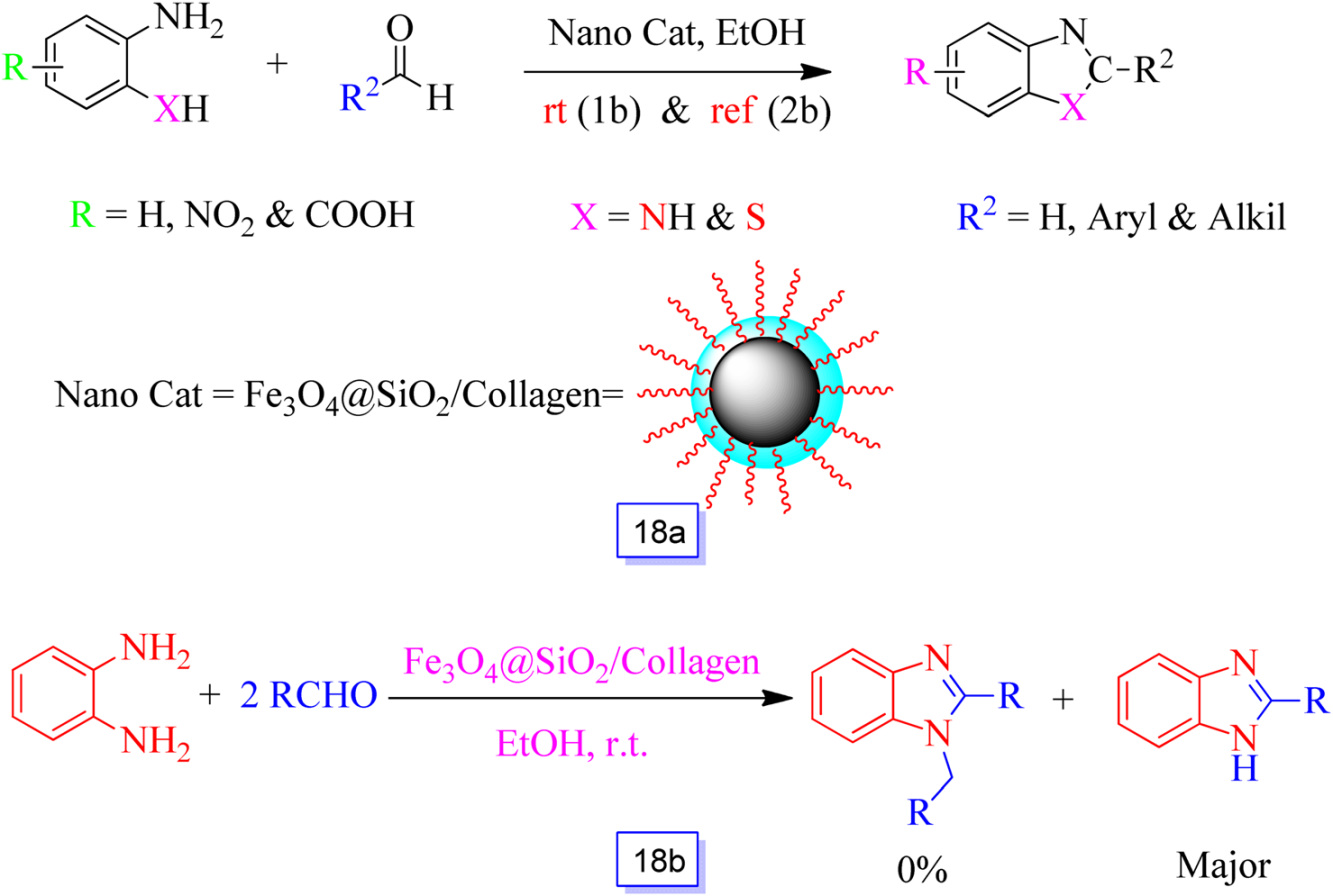
General procedure for the synthesis of benzimidazole (X:N) and benzothiazole (X:S) in the presence of Fe_3_O_4_@SiO_2_/collagen in ethanol solvent under reflux conditions (a), and selectivity of Fe_3_O_4_@SiO_2_/collagen in the synthesis of benzimidazole derivatives in ethanol solvent at room temperature (b).

Fe_3_O_4_@SiO_2_/collagen as a new magnetic nanocatalyst for the preparation of benzothiazole and benzimidazole derivatives in ethanol was studied by Ghafuri and coworkers^110^ (Scheme 18a). The synthesized Fe_3_O_4_@SiO_2_ was connected to collagen for the preparation of Fe_3_O_4_@SiO_2_/collagen. Collagen in Fe_3_O_4_@SiO_2_/collagen nanocatalyst has various functional groups that can create hydrogen bonds with protic compounds. Moreover, organic chains of collagen fibers can react with aprotic parts of organic compounds. This property has a principal role in the reaction times and yields in interaction with collagen existing in the nanocatalyst.^111–113^

One of the most important features of this nanocomposite is its unique properties and excellent selectivity (Scheme 18b). These nanocatalysts were magnetically isolated from the reaction media and reused for several continuous runs without a significant decrease in catalytic efficiency.

**Scheme 19:**
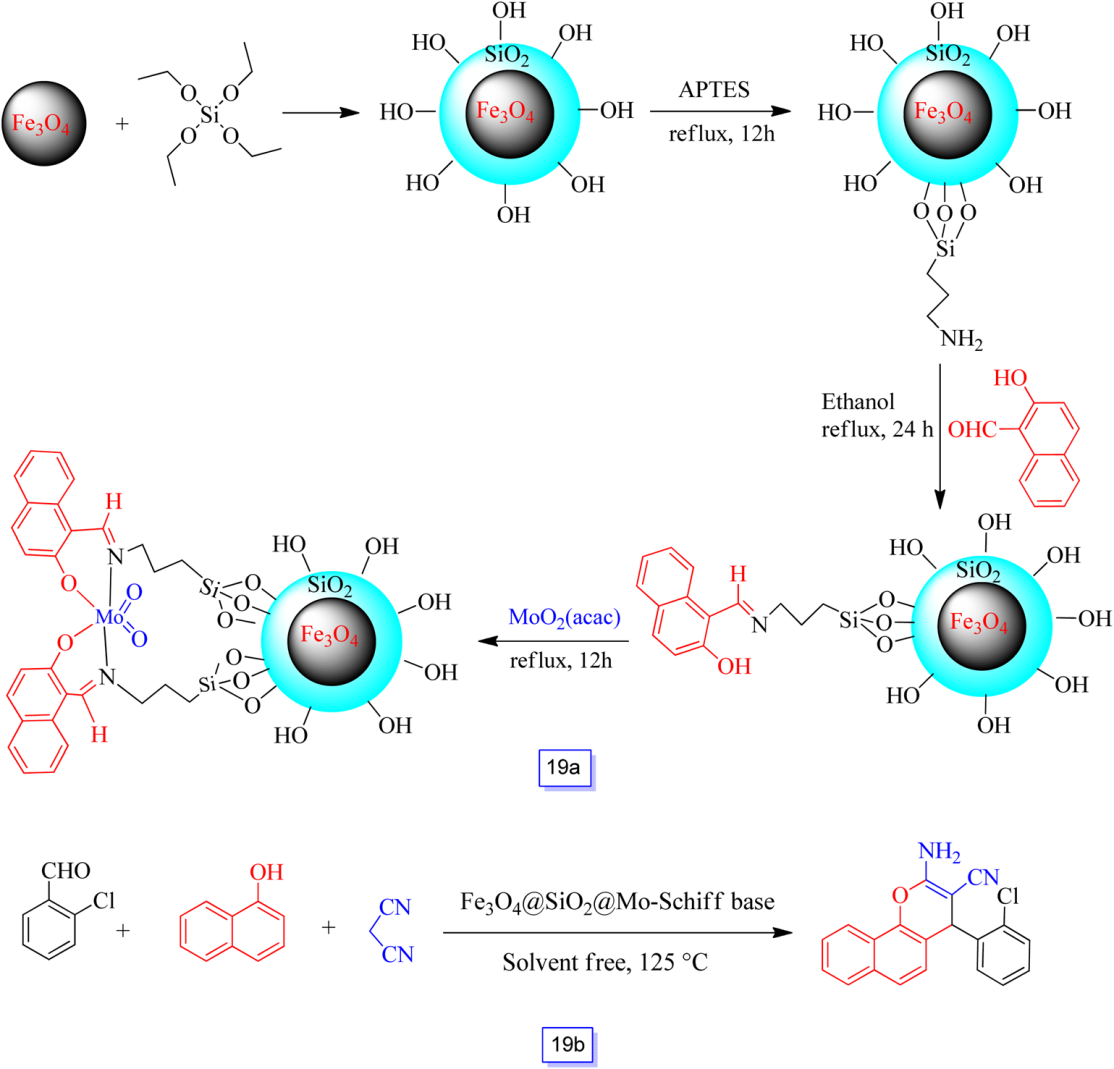
Preparation of Fe_3_O_4_@SiO_2_@Mo-Schiff base (a), and synthesis of 2-amino-4*H*-benzo[*h*]chromenes in the presence of Fe_3_O_4_@SiO_2_@Mo-Schiff base at 125 °C under solvent free conditions (b).

Monadi *et al.*^114^ reported a new procedure for the covalent attachment of a molybdenum Schiff base complex on the surface of silica covered with nanoparticles (Fe_3_O_4_@SiO_2_) (Scheme 19a).

In addition, the catalytic activity of Fe_3_O_4_@SiO_2_@Mo-Schiff base was investigated *via* three-component reactions between various substituted aldehydes, 1-naphthol, and malononitrile in one-pot synthesis to afford the corresponding 2-amino-4*H*-benzo[*h*]chromenes using Fe_3_O_4_@SiO_2_@Mo-Schiff base nanocatalyst under solvent-free and moderate conditions in good yields (Scheme 19b). After the reaction was completed, the nanocatalyst was collected from the reaction media by an external magnetic field. The nanoparticles were separated and washed with water and ethanol several times and reused for four cycles without a significant decrease in catalytic performance.

**Scheme 20:**
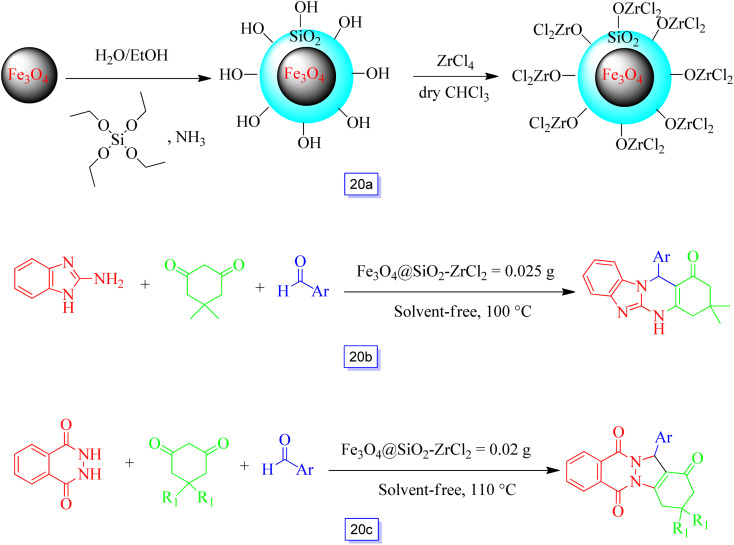
The preparation of Fe_3_O_4_@SiO_2_–ZrCl_2_-MNPs (a), Fe_3_O_4_@SiO_2_–ZrCl_2_-MNP catalyzed synthesis of tetrahydrobenzimidazo[2,1-*b*]quinazolin-1(2*H*)-one derivatives at 100 °C under solvent free conditions (b), and the Fe_3_O_4_@SiO_2_–ZrCl_2_-MNP catalyzed synthesis of 2*H*-indazolo[2,1-*b*]phthalazine-trione derivatives at 110 °C under solvent free conditions (c).

Shirini and colleagues^115^ prepared an environmentally beneficial and re-utilizable solid acid catalyst by immobilizing ZrCl_2_ onto silica-coated ferrite nanoparticles (Scheme 20a) for the efficient promotion of this nanocatalyst in solvent-free conditions for the preparation of tetrahydrobenzimidazo[2,1-*b*]quinazolin-1(2*H*)-ones and 2*H*-indazolo[2,1-*b*]phthalazine-triones (Scheme 20b and c). The prepared nanocatalyst was separated by magnetic isolation and reutilized for eight runs with just a small loss of performance, meaning that the procedure was practical and provided good yields.

**Scheme 21:**
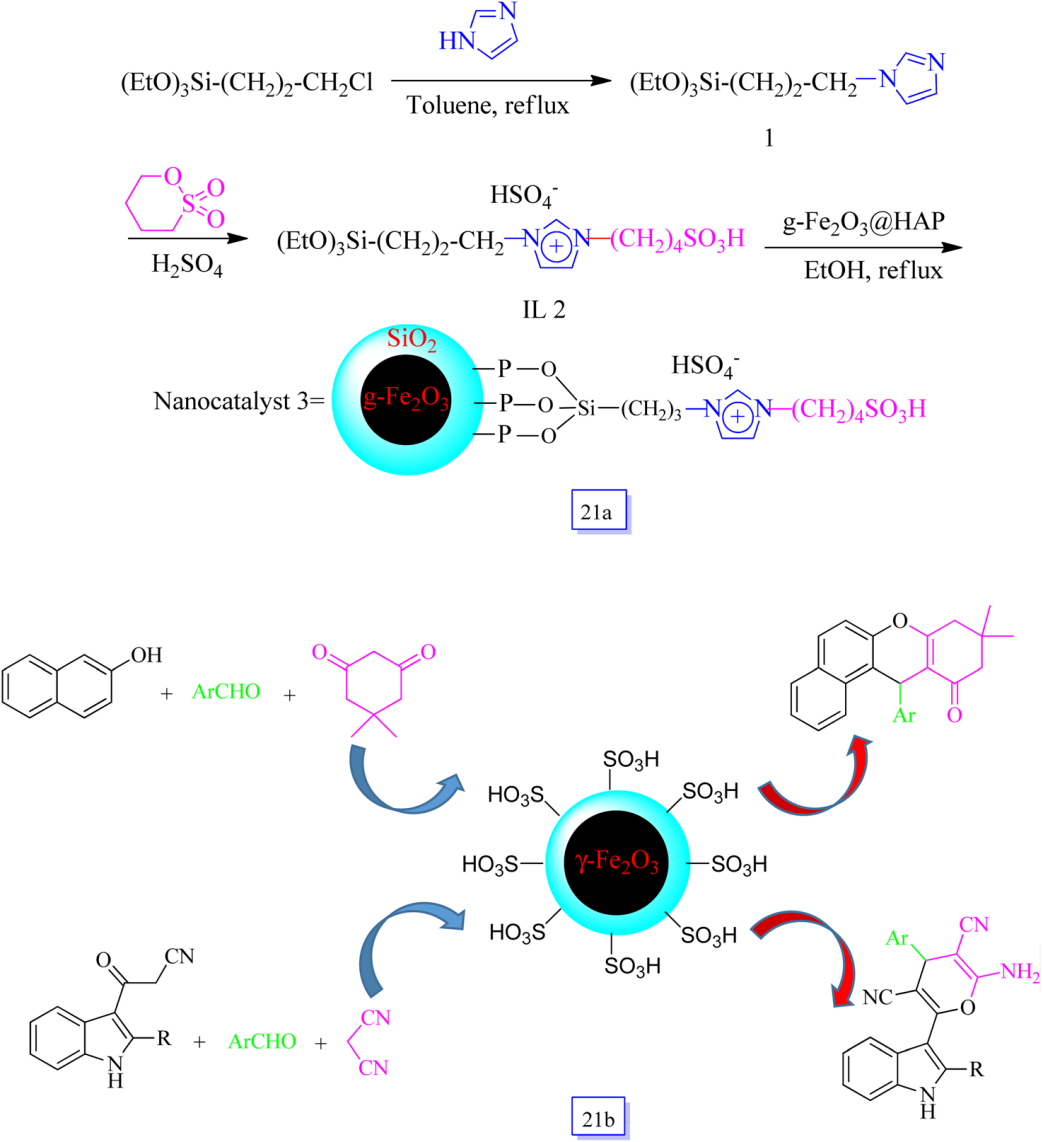
Synthesis of [Fe_2_O_3_@HAp]-supported dual acidic nanocatalyst (a), and synthesis of benzoxanthenones and 3-pyranylindoles in the presence of [Fe_2_O_3_@HAp]-supported dual acidic nanocatalyst 3 (b).

Manouchehr Mamaghani *et al.*^116^ reported a new HAp-encapsulated γ-Fe_2_O_3_-supported, as a dual acidic heterogeneous, re-utilizable, and very effective (Scheme 21a) catalyst. Its application for the one-pot synthesis of 3-pyranylindole and benzoxanthenone derivatives through three-component reactions (Scheme 21b). In this procedure, the utilization of the nanocatalyst presented a beneficial, green, and fast process to fabricate products in shorter reaction times (4–20 min) and good yields (87–96%). The paramagnetic property of this nanocatalyst enabled an easy, facile handling and simple procedure for the isolation of the catalyst by employing an external magnetic field, and it could be applied in 8 runs without a considerable decrease in catalytic performance.

**Scheme 22:**
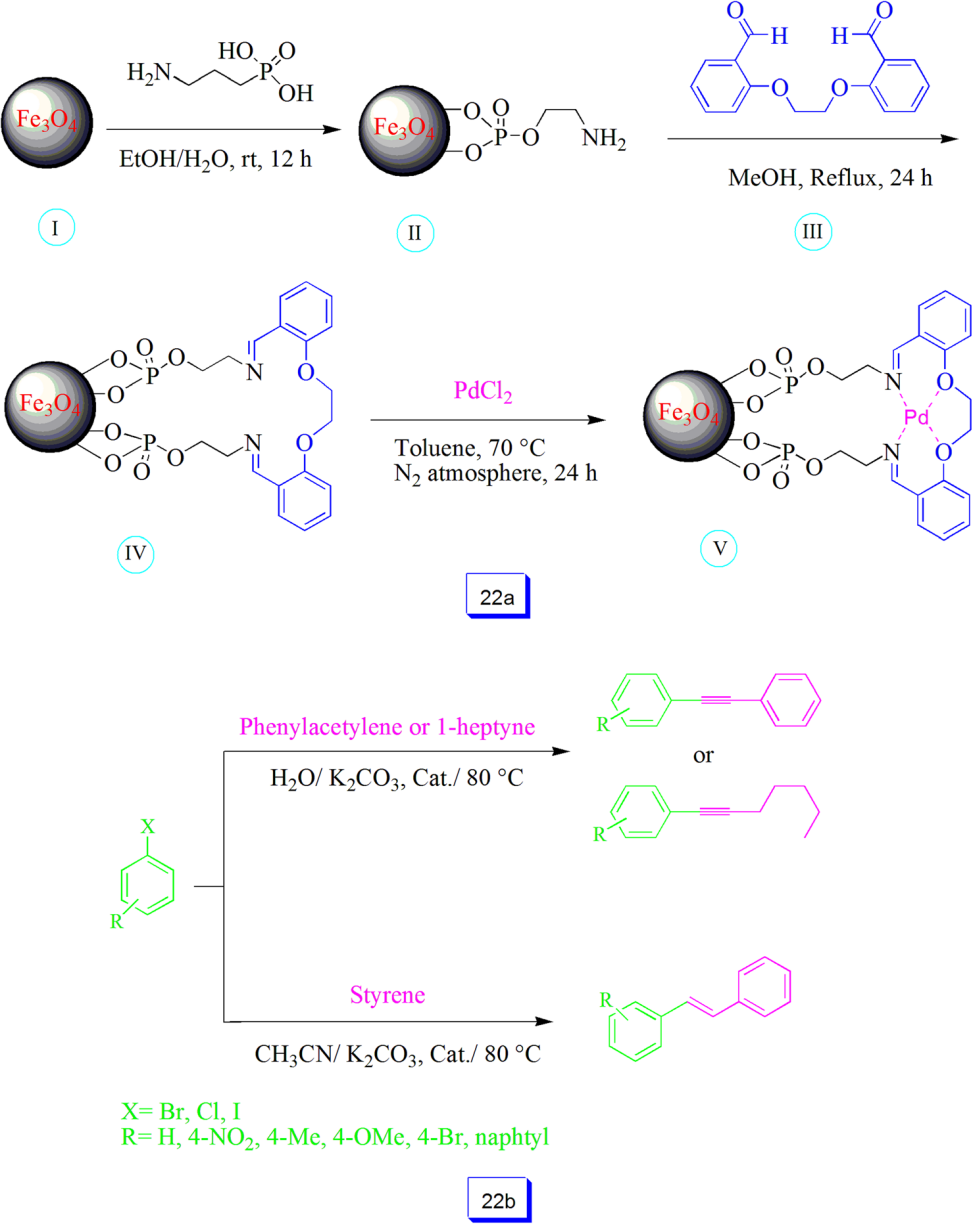
Preparation of novel task-specific nano-magnetic Schiff base ligand with phosphate spacer and its Pd nano-magnetic heterogeneous catalyst (a), and applicability of the novel Pd-based catalyst in C–C coupling reactions at 80 °C (b).

Mohammad Ali Zolfigol *et al.*^117^ prepared a new and task-specific Schiff base ligand connected to 2-aminoethyl dihydrogen phosphate as a spacer immobilized on the Fe_3_O_4_ nanoparticle surface (Scheme 22a). The resulting nanostructure was successfully applied as a Pd-supported nanocatalyst for Sonogashira and Mizoroki–Heck reactions (Scheme 22b). Therefore, this is the first study of the preparation and uses of Fe_3_O_4_@O_2_PO_2_(CH_2_)_2_NH_2_ nanoparticles as an appropriate spacer for the synthesis of an adjustable Schiff base ligand and its corresponding Pd complex.

**Scheme 23:**
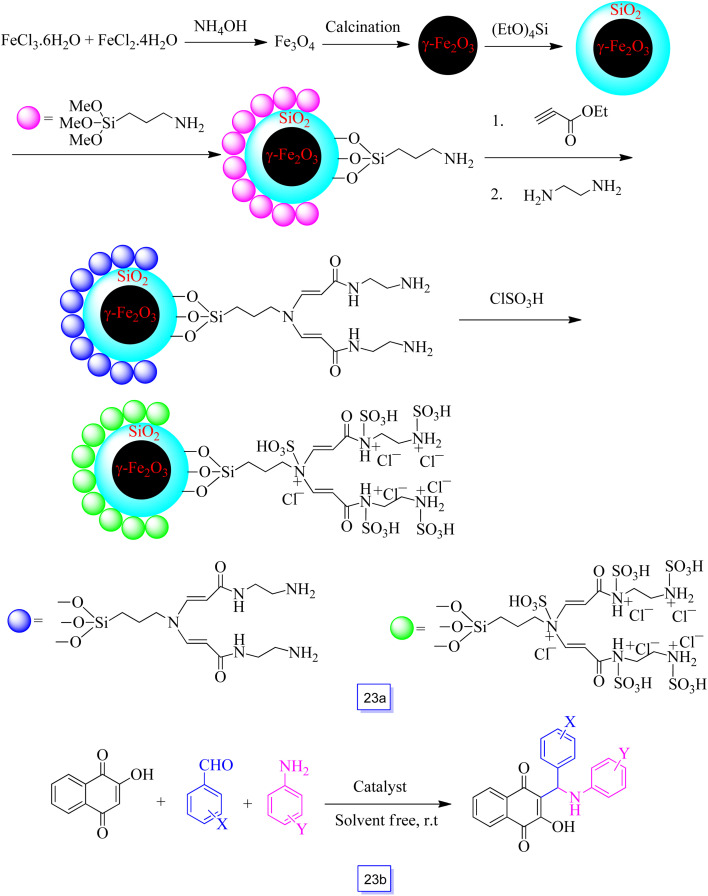
Synthesis of novel dendrimer super acidic magnetic nanoparticles as catalyst (a), and one-pot synthesis of hydroxyl naphthalene-1,4-dione derivatives at 125 °C under solvent free conditions (b).

Hamid Reza Shaterian *et al.*^118^ successfully prepared a new magnetic and super acidic nanostructure, *i.e.*, γ-Fe_2_O_3_@SiO_2_ functionalized with the dendrimer sulfonic acid as a new reusable and heterogeneous nanocatalyst (Scheme 23a). They have investigated the catalytic efficiency of the catalyst for efficient, facile, and one-pot preparation of 2-hydroxy-1,4-naphthoquinone derivatives through a three-component reaction of aromatic aldehydes, 2-hydroxynaphthalene-1,4-dione, and aniline derivatives (Scheme 23b). The advantages of this study are excellent yields, waste-free, low reaction time, room temperature, solvent-free, and mild conditions.

**Scheme 24:**
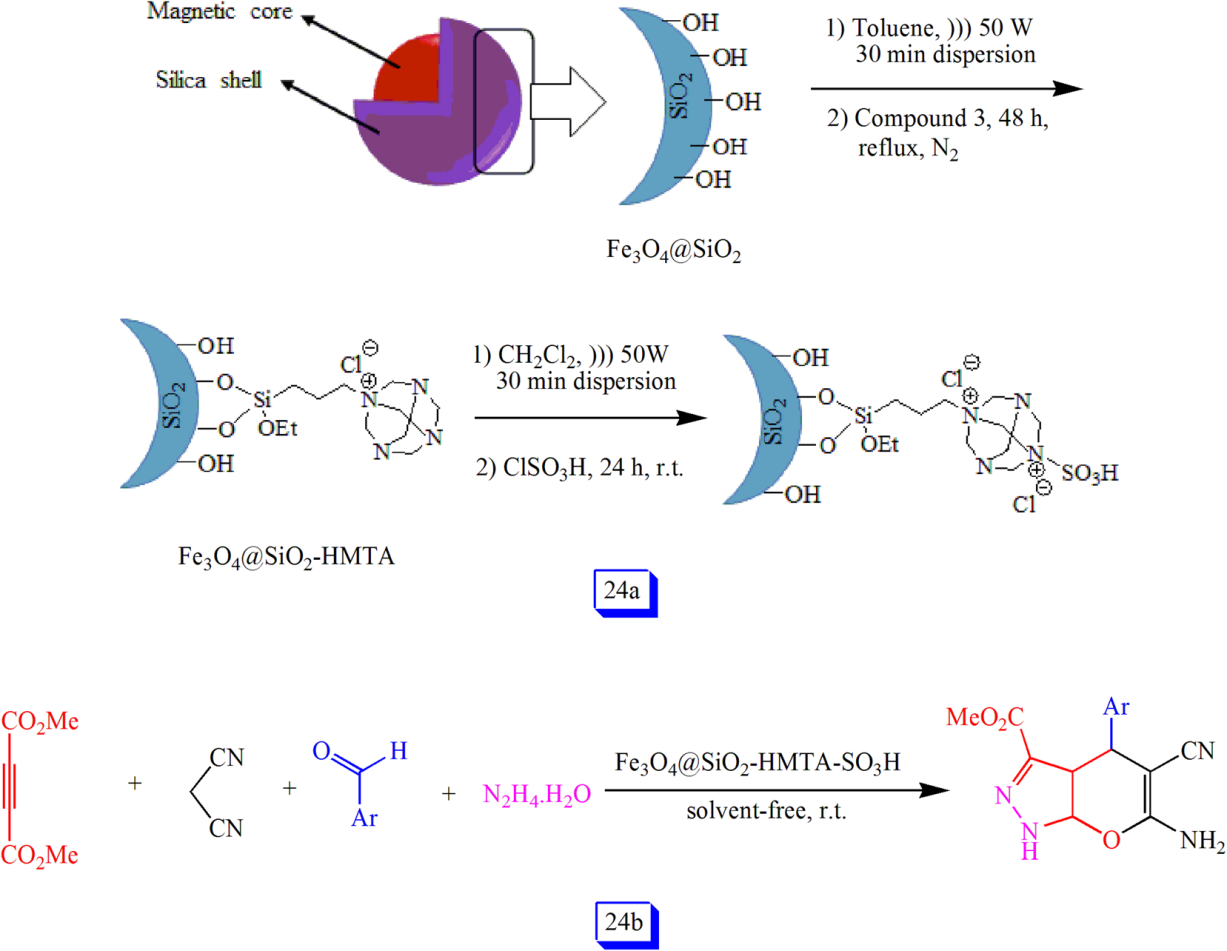
Preparation of Fe_3_O_4_@SiO_2_-HMTA-SO_3_H MNPs (a), and preparation of pyranopyrazole derivatives in the presence of Fe_3_O_4_@SiO_2_-HMTA-SO_3_H at room temperature under solvent free conditions (b).

Ramin Ghorbani-Vaghei *et al.*^119^ prepared a novel magnetic catalyst *via* the reaction of silanol groups (Si–OH), on the Fe_3_O_4_@SiO_2_ nanoparticles surface, with (3-chloropropyl) triethoxysilane followed by hexamethylenetetramine (HMTA) and then chlorosulfonic acid (Scheme 24a). Its catalytic performance was studied in the preparation of pyranopyrazole compounds (Scheme 24b). The products were produced in good to excellent yields within quick times under quite green conditions. Therefore, due to the simple and inexpensive method for the synthesis of this nanocatalyst and several other features, including high catalytic performance, easy isolation by applying a magnetic field, and good recoverability, it may have bright prospects in organic synthesis.

**Scheme 25:**
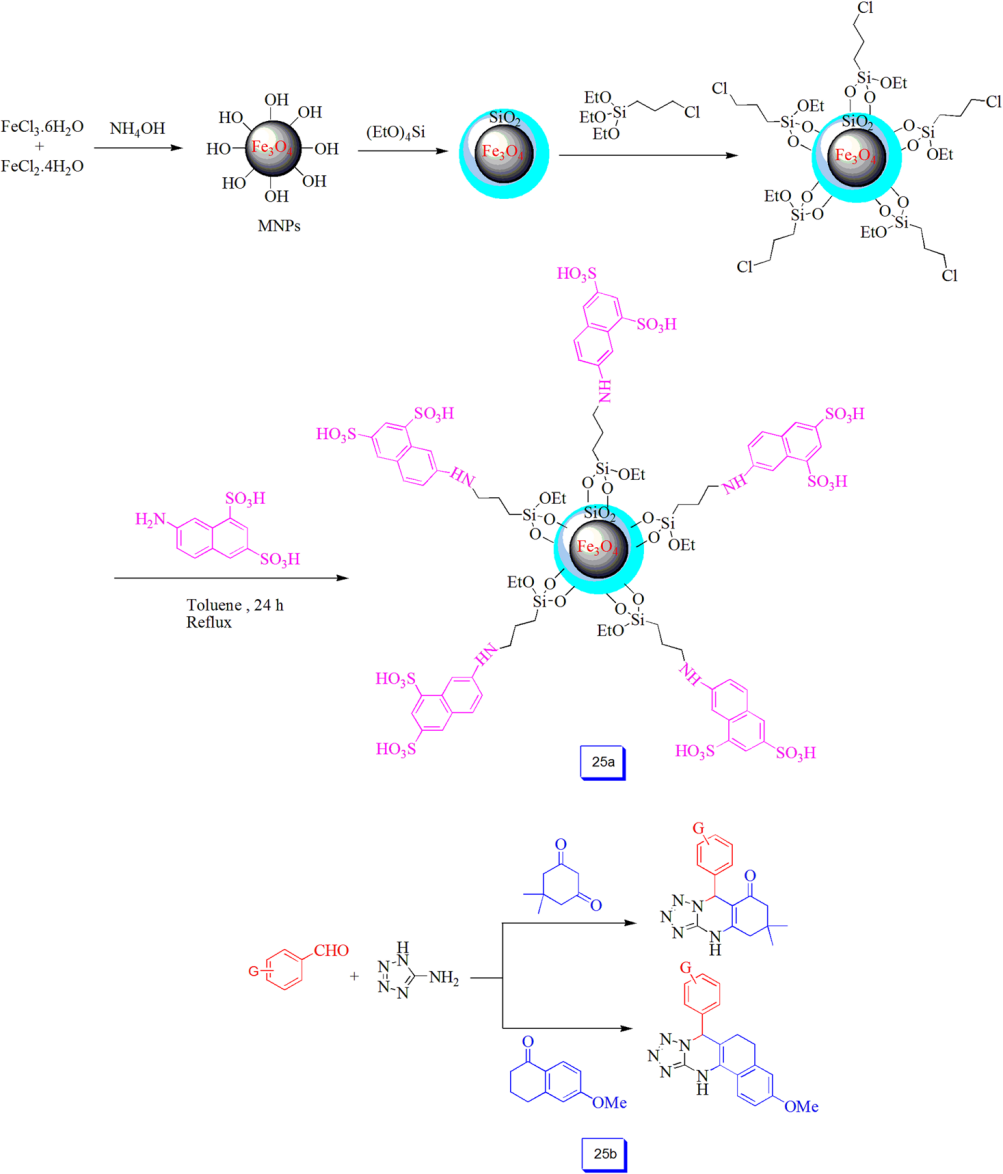
Stepwise synthesis pathway of Fe_3_O_4_@SiO_2_@propyl-ANDSA catalyst (a), and one-pot synthesis of tetrahydrotetrazolo[1,5-*a*]quinazolines and tetrahydrobenzo[*h*]tetrazolo[5,1-*b*]quinazolines (b).

Ramin Ghorbani-Vaghei *et al.*^120^ reported the immobilization of 7-aminonaphthalene-1,3-disulfonic acid (ANDSA) on Fe_3_O_4_@SiO_2_ nanostructure surface (Fe_3_O_4_@SiO_2_@propyl-ANDSA) (Scheme 25a). Then, its catalytic performance was studied in the preparation of derivatives of tetrahydrobenzo[*h*]tetrazolo[5,1-*b*]quinazolines and tetrahydrotetrazolo[1,5-*a*]quinazolines using the one-pot reaction of 5-aminotetrazole, aldehydes, and dimedone or 6-methoxy-3,4-dihyronaphthalen-1(2*H*)-one in H_2_O/EtOH at 100 °C (Scheme 25b). High product yields, simple purification, short reaction times, recoverability of the nanocatalyst, and simple procedure are the several advantages. There are two SO_3_H groups in the catalyst, which presented efficient acidic sites, resulting in the high performance of the nanocatalyst.

**Scheme 26:**
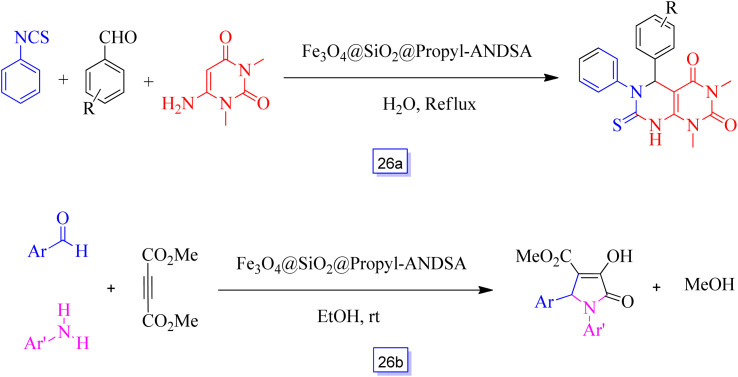
Fe_3_O_4_@SiO_2_@propyl-ANDSA catalyzed synthesis of pyrimido[4,5-*d*]pyrimidine in H_2_O solvent under reflux condition (a) and general synthesis of polysubstituted 3-hydroxy-2-pyrrolidinones in the presence of Fe_3_O_4_@SiO_2_@propyl-ANDSA and ethanol solvent at room temperature (b).

Also, Ramin Ghorbani-Vaghei *et al.*,^121^ using the nanocatalyst mentioned above (Scheme 25a), described a new, easy, effective, one-pot three-component method. This procedure prepared the substituted pyrimido[4,5-*d*]pyrimidines through the reaction of isothiocyanate, *N*,*N*-dimethyl-6-amino uracil, and aromatic aldehydes using water as a green solvent and without applying any other toxic organic agents (Scheme 26a). In comparison with other methods, using these hybrid inorganic–organic heterogeneous catalysts can assist in obtaining a green approach, high catalytic efficiency, simple recovery with a magnetic field, and quick reaction times. Ghorbani-Vaghei *et al.*^122^ prepared 7-aminonaphthalene-1,3-disulfonic acid immobilized on Fe_3_O_4_ nanoparticle surface as a nanocatalyst for the one-pot and multi-component synthesis of substituted 3-pyrrolin-2-ones without the usage of any toxic organic compounds (Scheme 26b). Excellent catalytic efficiency, simple recovery, and ability to be reutilized several times without considerable loss of its catalytic performance using a magnetic field are the environmentally-friendly features of this catalytic method.

**Scheme 27:**
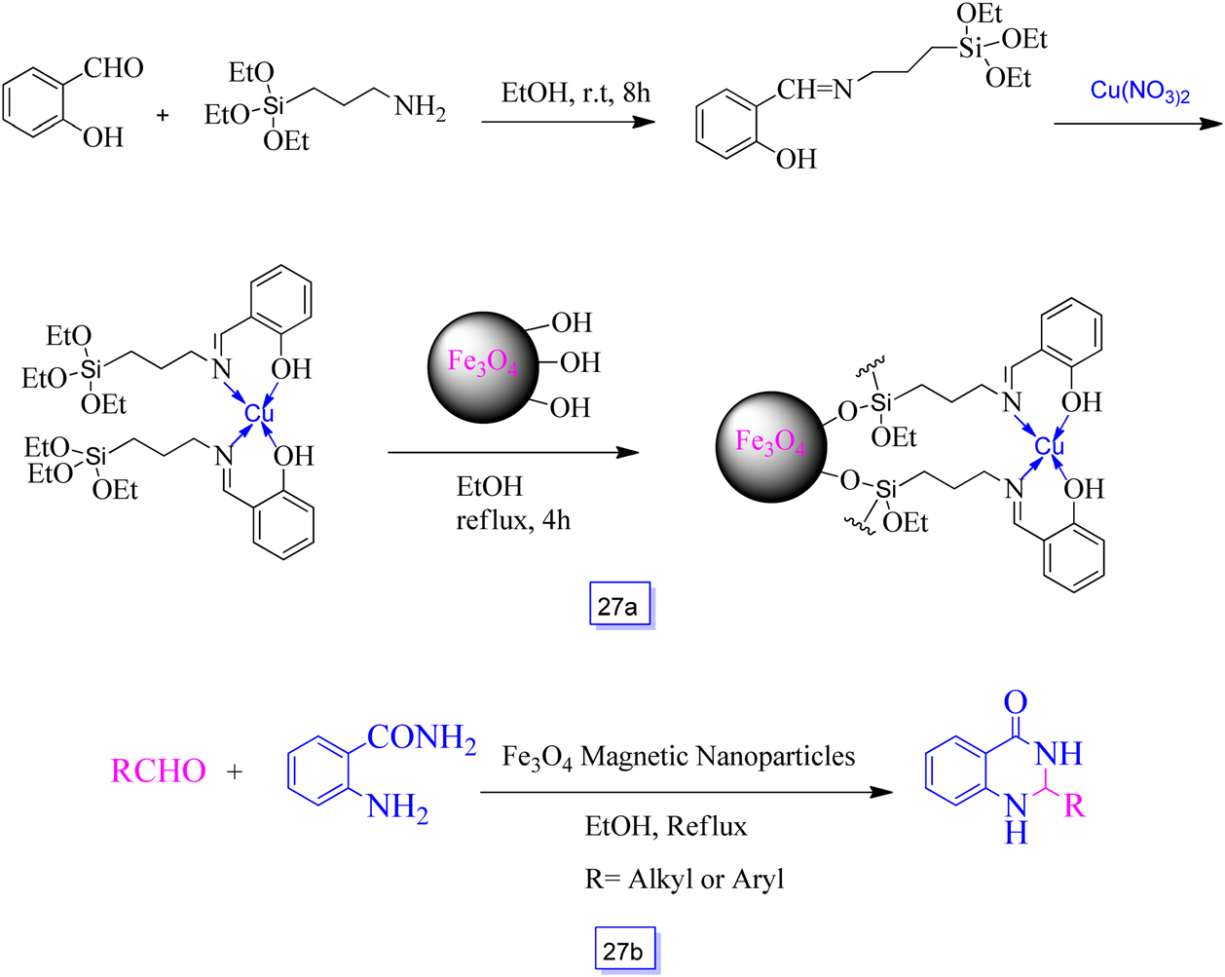
Synthesis of Fe_3_O_4_–Schiff base of Cu(ii) (a) and synthesis of 2,3-dihydroquinazolin-4(1*H*)-ones in the presence of Fe_3_O_4_ magnetic nanoparticles and ethanol solvent at reflux conditions (b).

Arash Ghorbani-Choghamarani *et al.*^123^ reported Fe_3_O_4_–Schiff base of Cu(ii) (Scheme 27a) as a reusable and heterogeneous nanocatalyst for the fast and effective synthesis of several 2,3-dihydroquinazolin-4(1*H*)-one derivatives from the condensation of two-component reaction of aldehyde and 2-aminobenzamide (Scheme 27b). This procedure is easy, green, and inexpensive. Isolation and recovery can also be efficiently performed with magnetic separation of the Fe_3_O_4_ nanoparticles.

**Scheme 28:**
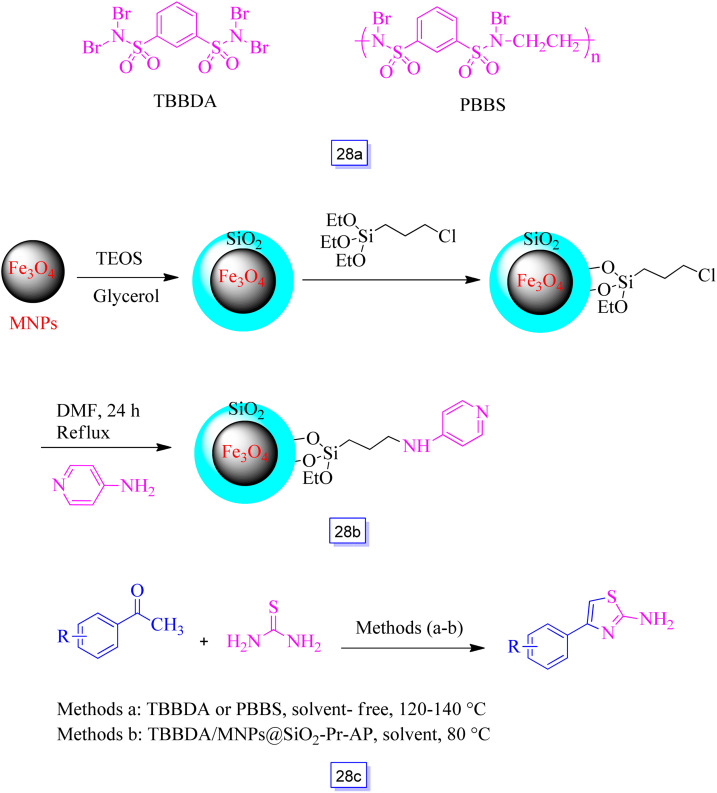
The synthetic route of the nanocatalyst and the structure of TBBDA and PBBS (a), synthetic route of nanocatalyst (b), and one-pot synthesis of 2-amino-4-arylthiazole derivatives (c).

Ramin Ghorbani-Vaghei *et al.*^124^ reported three separate types of catalysts, *i.e.*, poly(*N*,*N*′-dibromo-*N*-ethylbenzene-1,3-disulfonamide) [PBBS], *N*,*N*,*N*′,*N*′-tetrabromobenzene-1,3-disulfonamide [TBBDA] (Scheme 28a), and a combination of TBBDA and Fe_3_O_4_ nanoparticles functionalized with 4-amino-pyridine supported on silica surface (MNPs@SiO_2_-Pr-AP) (Scheme 28b). This catalyst was used for the preparation of 2-amino-4-aryl thiazole derivatives *via* the reaction of thiourea and substituted acetophenones (Scheme 28c). The experiments exhibited that the application of TBBDA along with MNPs@SiO_2_-Pr-AP provides high yields of the products in the quickest reaction time.

**Scheme 29:**
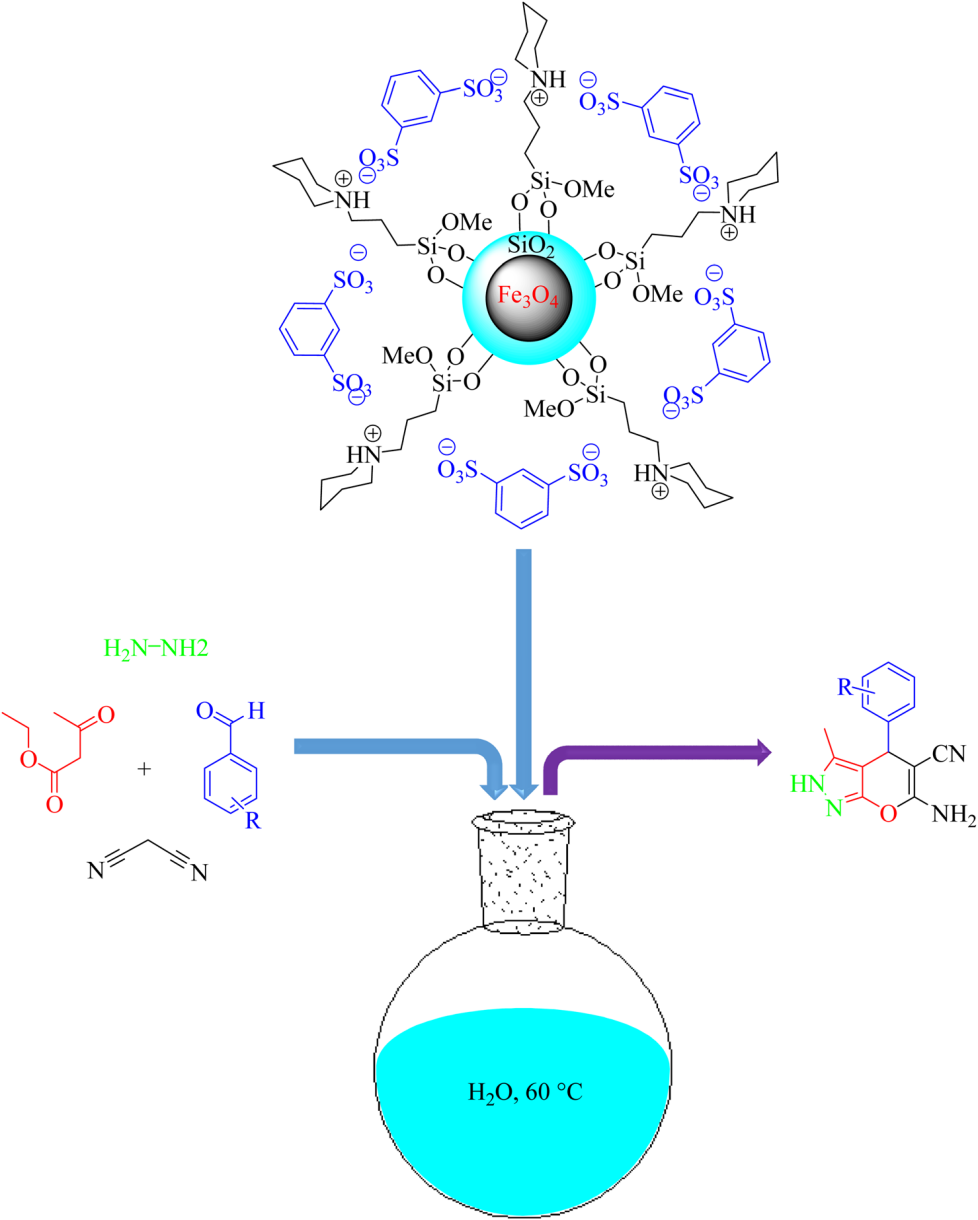
Synthesis of 6-amino-4-(4-methoxyphenyl)-5-cyano-3-methyl-1-phenyl-1,4-dihydropyrano[2,3-*c*]pyrazoles using Fe_3_O_4_@SiO_2_@piperidinium benzene-1,3-disulfonate as a catalyst in the presence of H_2_O solvent at 60 °C.

Ramin Ghorbani-Vaghei *et al.*^125^ reported Fe_3_O_4_ functionalized with piperidinium benzene-1,3-disulfonate as an easy, green, and effective nanocatalyst for the one-pot preparation of pyrano[2,3-*c*]pyrazole derivatives obtained from the four component reaction between malononitrile, aryl aldehydes, ethyl acetoacetate, and hydrazine hydrate in water at 60 °C (Scheme 29). The Fe_3_O_4_@SiO_2_ nanoparticle supported IL was prepared. This procedure provided benefits such as less reaction time, clean reaction, simple purification, good to high yields, and easily recyclable catalyst.

## 5. Conclusion

During the past few decades, studies on various kinds of magnetic nanostructured catalysts and their utilization in different organic reactions have achieved many successes. Nanocatalysis with magnetic reusability is a quickly growing field in the context of the high requirements for the progress of green chemistry. Many magnetically recyclable catalysts were utilized in various reactions, including Sonogashira, Heck, Suzuki, Hiyama, hydrogenation, alkyne–azide cycloaddition, oxidation, reduction, epoxidation of alkenes, arylation, alkylation, Fenton-like reaction, and multicomponent “one-pot” synthesis. To avoid aggregation and obtain grafting catalyst varieties on prepared MNPs, functionalization and modification of MNPs with immobilizing ligands or encapsulating/coating substances (such as silica, small molecules, carbon, polymers, mesoporous materials, ionic liquids, carbon nanotubes, and graphene) are necessary. The advantages of using these MNPs as catalysts include good to excellent yields of the products, simple work-up procedure, quick reaction times, and recyclability of the nanocatalysts in most cases. This particular review only covers a segment of the applications of magnetic nanomaterials as efficient catalysts in the field of different multicomponent reactions. Nevertheless, they have tremendous potential in several other fields, including biomedical therapeutics and biotechnology. In the future, we will be focusing on unveiling the bio applications of these diverse magnetic nanomaterials.

## Conflicts of interest

There are no conflicts to declare.

## Supplementary Material
